# Supplementary material for: Cohort profile: the Oxford Parkinson’s Disease Centre Discovery Cohort MRI substudy (OPDC-MRI)
Source: BMJ Open. 2020 Aug 13;10(8):e034110. doi: 10.1136/bmjopen-2019-034110 (PMC7430482; doi:10.1136/bmjopen-2019-034110)
Supplement: Supplementary data [file bmjopen-2019-034110supp001.pdf]

Supplementary material - Cohort Profile: the Oxford Parkinson's Disease Centre Discovery Cohort Magnetic Resonance Imaging sub-study (OPDC-MRI).

Griffanti et al.

Supplementary Table S1

Questionnaires administered to the OPDC Discovery cohort participants and derived variables.

Patient-Completed Questionnaires	
**Demographics**	Basic demographic information
**Big Five Inventory**[1]	Personality profiling
**Epworth Sleepiness Scale**[2]	Daytime sleepiness questionnaire
Sleepiness, (%)	dichotomised to 'unusual level of sleepiness - ESS >=11' versus 'normal results - EPS <=10'
**RBD Questionnaire**[3]	Patient reported features of RBD
RBD, (%)	dichotomised to 'positive result - RBD >=5' versus 'normal - RBD<=4'
**Health-Related Quality of Life EQ5D and EQ-VAS**[4]	General assessment of quality of life
Pain, EQ5D, (%)	pain score from EQ-5D health questionnaire dichotomised to 'no pain or discomfort' versus 'moderate or extreme pain or discomfort'
**Constipation Questionnaire**	Assessment of frequency of bowel movements
Constipation, (%)	average daily bowel movement frequency in the past week dichotomised to 'constipation - <1' versus 'normal - >=1'
Use of laxatives, (%)	use of laxatives in the past week dichotomised to 'any' versus 'none'
**UPDRS I, Non-motor aspects of daily living**[5]	Assessment of basic non-motor symptoms in PD in the last week. Each item was dichotomised as ‘present – score 1-4’ versus ‘absent – score 0’.
**UPDRS II, Motor aspects of daily living**[5]	Assessment of basic motor symptoms in PD in the last week. Each item was dichotomised as ‘present – score 1-4’ versus ‘absent – score 0’.
**Social background**	Information about education, wealth and employment.
Accommodation at diagnosis, (own, %)	Dichotomised to ‘own’ versus ‘renting’
Bedrooms at diagnosis, (≤3, %)	Dichotomised to ‘≤3’ versus ‘>3’
Vehicles at diagnosis, (≤1, %)	Dichotomised to ‘≤1’ versus >1’
Employment at diagnosis, (employed, %)	Dichotomised to 'employed in any capacity' versus not employed
Last job, (managing people, %)	Dichotomised to 'job involving managing people' versus 'job not involving managing people'
**Leeds Depression and Anxiety General Scales**[6]	Assessment of level of depression and anxiety in undiagnosed cases.

Depression, Leeds SAD General, (%)	dichotomised to 'case - >=7' versus 'non-case - <7'[6]
Anxiety, Leeds SAA General, (%)	dichotomised to 'case - >=7' versus 'non-case - <7'[6]
**Beck Depression Inventory (BDI)**[7]	Extensive assessment of symptoms of depression.
**Questionnaire for Impulsive-Compulsive Disorders in Parkinson's Disease (QUIP Anytime Short)**[8]	Assessment of impulsive and compulsive disorders in PD occurring anytime in life.
Any ICD, QUIP, (%)	dichotomised score for any main impulsive disorder (gambling, sex, buying or eating)
Any other OCD, QUIP, (%)	dichotomised score for any other compulsive behaviour (organised activities, ordering, purposeless walking or driving)
Overmedicating, QUIP, (%)	dichotomised score for any excessive parkinsonian medication use
Nurse-Administered Questionnaires	
**General Exam**	Weight, height, lying and standing blood pressure.
BMI	Body Mass Index calculated as: weight in kg divided by square of height in meters.
Orthostatic hypotension, (%)	Orthostatic hypotension defined as difference between lying and standing blood pressure greater than 20 (SBP) or 10 (DBP).
**MOCA**[9]	Assessment of general cognition. Additional point was added for patients with <=12 years of education.
Cognitive impairment, (normal:MCI:dementia, %)	Defined based on MOCA scores: normal cognition 26-30, MCI 21-25, dementia <=20[10-12]
**MMSE**[13]	Assessment of general cognition.
**Phonemic and Semantic Fluency**	Assessment of fluency scored according to population norms.
**Peg board tests**[14]	Test of manual dexterity. Test results indicate number of pegs/items inserted into peg board within 30s (1min for 'Arm coordination')
Arm asymmetry (absolute value of right score - left score), Peg board, (mean±SD)	absolute difference between right and left hand scores reflecting asymmetry of disease
**Get-up-and-go**[15]	Test of walking speed. The result is an average in seconds of three trials of timed 3m walk.
**Flamingo test** [16]	Test of balancing on one leg for 30s. The result is dichotomised to 'subjects who completed 30s trial' versus 'subjects who failed before 30s'.
Doctor Administered Questionnaires	
**Past Medical History**	Review of medical history covering most common conditions.
Vascular risk factors, (%)	Percentage of patients with any of the following: high blood pressure, high cholesterol level, diabetes mellitus
Cerebro-vascular disease, (%)	Percentage of patients with any of the following: stroke or TIA
Cardio-vascular disease, (%)	Percentage of patients with any of the following: angina, heart attack, heart failure

Cancers, (%)	Percentage of patients with any of the following: lung, bowel, breast, prostate, melanoma, or any other cancer reported by patient
Respiratory disease, (%)	Percentage of patients with any of the following: asthma, chronic bronchitis, emphysema
**Mini Environmental Risk Questionnaire for PD Baseline (MERQ-PD B)**[17]	Assessment for exposure to commonly accepted risk factors for PD.
Smoking before PD, (%)	Dichotomised to 'smoking at least one cigarette per day for at least 6 months' versus 'non-smoker by that definition'.
Alcohol consumption before PD, (%)	Dichotomised to 'drinking any alcohol' versus 'non-drinker'.
Medication (different types) before PD	Dichotomised to 'any use' versus 'no use'.
**Modified Schwab & England Activities of Daily Living**	Assessment of activities of daily living.
**PD Features**[17]	Presence of typical features of PD at diagnosis as reported by the patient and in the notes.
**Family History**[17]	Extensive family history including occurrence of PD and other neurological disorders in the family.
**PD Medication**	List of current and previous medication taken for PD.
Levodopa equivalent daily dose (LEDD)[18]	Calculated from all current dopaminergic medication.
**Clinical Global Impression of Change (CGI)**	Eight-level scale assessing change in symptoms after starting PD medication.
**Other Medication**[17]	List of medication unrelated to PD.
**Sniffin Sticks**[19]	Assessment of hyposmia. Sniffin Sticks was score dichotomised at 10th percentile according to normal values for age and sex.
**UPDRS I Non-motor features of daily living**[5]	Clinician-assessed non-motor symptoms of PD.
Fatigue in PD, (%)	Dichotomised score from item 1.13 of MDS-UPDRS I: 'any fatigue' versus 'no fatigue'
Apathy in PD, (%)	Dichotomised score from item 1.5 of MDS-UPDRS I: 'any apathy' versus 'no apathy'
Hallucinations in PD, (%)	Dichotomised score from item 1.2 of MDS-UPDRS I: 'any hallucinations' versus 'no hallucinations'
**UPDRS III Motor Examination**[5]	Standardised assessment of PD signs.
Rigidity score (score/number of items)	Sum of scores from item 3.3 MDS-UPDRS III divided by 5
Bradykinesia scores, (score/number of items)	sum of scores from items 3.4, 3.5, 3.6, 3.7, 3.8, divided by 10
Postural scores, (score/number of items)	Sum of scores from items 3.9, 3.10, 3.11, 3.12, 3.13, divided by 5

Tremor scores, (score/number of items)	Sum of scores from items 3.15, 3.16, 3.17, divided by 9
Motor subtype, (PIGD:Mix:Tremor, %)	Sum of scores from all MDS-UPDRS tremor items (2.10, 3.15, 3.16, 3.17) divided by the sum of the postural and gait items (2.12, 2.13, 3.10, 3.11, 3.12) gives a Tremor/PIGD index; index <=1 defines PIGD subtype, >=1.5 - tremor dominant subtype, between 1-1.5 - mixed subtype[20]
Face and neck score, (score/number of items)	Sum of scores from MDS-UPDRS III items for face and neck symptoms (3.1, 3.2, 3.3 Neck, 3.17 Lip/Jaw) divided by 4
Arms score, (score/number of items)	Sum of scores from MDS-UPDRS III items for upper extremities (3.3 RUE, 3.3 LUE, 3.4, 3.5, 3.6, 3.15, 3.16, 3.17 RUE, 3.17 LUE) divided by 14
Legs score, (score/number of items)	Sum of scores from MDS-UPDRS III items for lower extremities (3.3 RLE, 3.3 LLE, 3.7, 3.8, 3.17 RLE, 3.17 LLE) divided by 8
Symptom laterality, (right : left : symmetrical, %)	Right-dominant symptoms: difference between all right-sided scores and all left-sided scores >=4; left-dominant symptoms: difference between all left-sided scores and all right-sided scores >=4; symmetrical: intermediate scores[21]
Annualised UPDRS III (UPDRS III/disease duration)	Total UPDRS III score divided by disease duration in years since symptom onset, reflecting symptomatic progression since disease onset
**UPDRS IV Motor Complications**[5]	Assessment of dyskinesia, motor fluctuations and dystonia.
Presence of Dyskinesia, (%)	Dichotomised score from item 4.1 of MDS-UPDRS IV: 'No dyskinesia' versus 'Any dyskinesia'
Presence of Motor Fluctuations, (%)	Dichotomised score from item 4.3 of MDS-UPDRS IV: 'No OFF time' versus 'Any OFF time'
**Specific Clinical Features**[17]	Assessment of atypical parkinsonian features not fitting with idiopathic PD.
Sexual dysfunction, (%)	Significant change in sexual functioning in the past year
Erectile dysfunction, (%)	Ability to maintain erection without treatment dichotomised to 'very poor or poor' versus 'fair, good or very good'
**Likelihood of Diagnosis**	Clinician-assessed likelihood of diagnosis of idiopathic PD from 0%-100%.

References for Supplementary Table S1

1. John, O. P. & Srivastava, S. The Big Five trait taxonomy: History, measurement, and theoretical perspectives. *Handbook of personality: Theory and research* **2,** 102–138 (1999).
2. Johns, M. W. A new method for measuring daytime sleepiness: the Epworth sleepiness scale. *Sleep* **14,** 540–545 (1991).
3. Stiasny-Kolster, K. *et al.* The REM sleep behavior disorder screening questionnaire--a

new diagnostic instrument. *Mov Disord* **22,** 2386–2393 (2007).
4. EuroQol--a new facility for the measurement of health-related quality of life. The EuroQol Group. *Health Policy* **16,** 199–208 (1990).
5. Goetz, C. G. *et al.* Movement Disorder Society-sponsored revision of the Unified Parkinson's Disease Rating Scale (MDS-UPDRS): Process, format, and clinimetric testing plan. *Mov Disord* **22,** 41–47 (2007).
6. Snaith, R. P., Bridge, G. W. & Hamilton, M. The Leeds scales for the self-assessment of anxiety and depression. *Br J Psychiatry* **128,** 156–165 (1976).
7. Beck, A., Ward, C. & Mendelson, M. Beck depression inventory (BDI). *Archives of General Psychiatry* (1961).
8. Weintraub, D. *et al.* Validation of the questionnaire for impulsive-compulsive disorders in Parkinson's disease. *Mov Disord* **24,** 1461–1467 (2009).
9. Nasreddine, Z. S. *et al.* The Montreal Cognitive Assessment, MoCA: a brief screening tool for mild cognitive impairment. *J Am Geriatr Soc* **53,** 695–699 (2005).
10. Dalrymple-Alford, J. C. *et al.* The MoCA: Well-suited screen for cognitive impairment in Parkinson disease. *Neurology* **75,** 1717–1725 (2010).
11. Gill, D. J., Freshman, A., Blender, J. A. & Ravina, B. The Montreal cognitive assessment as a screening tool for cognitive impairment in Parkinson's disease. *Mov Disord* **23,** 1043–1046 (2008).
12. Nazem, S. *et al.* Montreal cognitive assessment performance in patients with Parkinson's disease with “normal” global cognition according to mini-mental state examination score. *J Am Geriatr Soc* **57,** 304–308 (2009).
13. Folstein, M. F., Folstein, S. E. & McHugh, P. R. ‘Mini-mental state’. A practical method for grading the cognitive state of patients for the clinician. *J Psychiatr Res* **12,** 189–198 (1975).
14. Desrosiers, J., Hébert, R., Bravo, G. & Dutil, E. The Purdue Pegboard Test: normative data for people aged 60 and over. *Disabil Rehabil* **17,** 217–224 (1995).
15. Shumway-Cook, A., Brauer, S. & Woollacott, M. Predicting the probability for falls in community-dwelling older adults using the Timed Up & Go Test. *Phys Ther* **80,** 896–903 (2000).
16. Tsigilis, N., Douda, H. & Tokmakidis, S. P. Test-retest reliability of the Eurofit test battery administered to university students. *Percept Mot Skills* **95,** 1295–1300–1300 (2002).
17. http://www.commondataelements.ninds.nih.gov
18. Tomlinson, C. L. *et al.* Systematic review of levodopa dose equivalency reporting in Parkinson's disease. *Mov Disord* **25,** 2649–2653 (2010).
19. Hummel, T., Kobal, G., Gudziol, H. & Mackay-Sim, A. Normative data for the ‘Sniffin' Sticks’ including tests of odor identification, odor discrimination, and olfactory thresholds: an upgrade based on a group of more than 3,000 subjects. *Eur Arch Otorhinolaryngol* **264,** 237–243 (2007).
20. Skeie, G. O., Muller, B., Haugarvoll, K., Larsen, J. P. & Tysnes, O. B. Differential effect of environmental risk factors on postural instability gait difficulties and tremor dominant Parkinson's disease. *Mov Disord* **25,** 1847–1852 (2010).
21. Uitti, R. J., Baba, Y., Whaley, N. R., Wszolek, Z. K. & Putzke, J. D. Parkinson disease: handedness predicts asymmetry. *Neurology* **64,** 1925–1930 (2005).

Supplementary Information

Discontinued sequences

Supplementary Table S2 illustrates the main acquisition parameters and the number of available subjects per group for each of the sequences that were acquired between 2010 and 2016.

The diffusion-weighted sequence with reduced FOV centered on the substantia nigra (SN) was inspired by the study by Vaillancourt and colleagues [1], who found reduced FA in the SN of subjects with Parkinson's compared with controls, especially in the caudal compared with the rostral region of interest. We acquired a similar sequence in our first 50 participants, but did not find any difference between Parkinson's and controls (unpublished). We therefore decided to discontinue the sequence.

DESPOT1 and DESPOT2 (driven equilibrium single pulse observation of T1and T2, respectively) are a set of rapid combined T1 and T2 mapping approaches developed by Deoni and colleagues to be acquired in a clinically acceptable time [2]. They also demonstrated that by acquiring DESPOT2 in addition to DESPOT1 it is possible to retrospectively calculate 'synthetic' weighted images with arbitrary T1 or T2 weighting, since the underlying relaxation parameters are known. A study using quantitative MRI (qMRI) in early Parkinson's [3] found widespread reduction of midbrain T1 values contralateral to the clinically more severely affected limbs and suggested that T1 may be a valuable marker for the monitoring of progressive neuronal loss in Parkinson's. T1 qMRI probes the chemical composition of the tissues, providing complementary information to the microstructural one available with diffusion imaging. In light of these findings we experimented with this sequence in OPDC-MRI. Quantitative T1 maps were generated using DESPOT-HiFi (dual SPGR, 144 slices) with two IR-SPGR images to assess B1 field inhomogeneity[2]. While we were able to use the T1 maps obtained from DESPOT1[4], the quality of DESPOT2 obtained was not deemed sufficient to perform further analyses.

The study by Baudrexel and colleagues[3] also acquired multi-echo T2* and performed T2* mapping of the midbrain, which revealed a bilateral decrease of T2* values restricted to the SN, indicating a local increase in total iron content. They conclude that nigral T2* reductions might be associated with an increased general vulnerability for the development of the disorder. We therefore included this small FOV, multi ECHO T2* sequence to explore its diagnostic usefulness given that SN

iron accumulation seems implicated in Parkinson's and T2* correlates with iron content.

This sequence was subsequently replaced with whole brain SWI because, despite not being able to provide T2* mapping (non multi-echo), it has a much shorter acquisition time and covers the whole brain, allowing, for example, to quantify iron content in the basal ganglia (see main text for details).

Table S2. Discontinued MRI experimental sequences: parameters used in the study and number of available datasets for each modality.

	**SN dMRI**	**qT1**	**qT2**	**SN GRE**
Sequence type	EPI	3D, DESPOT-HiFi	3D, DESPOT-HiFi	2D, T2*-weighted
Period of acquisition	2010-2013	2010-2014	2010-2014	2010-2016
TR (ms)	3000	8	55	648
TE (ms)	82	3.7	2.8	5 to 60#
TI (ms)	--	--	--	--
Flip angle (degrees)	90	4, 18	15, 60	45
Voxel size (mm)	0.8x0.8x4	1.1x1.1x1.1	1.1x1.1x1.1	1 x 1 x 2
FoV read (mm)	60	270	270	256
Fov phase (%)	41.7	83.3	83.3	100
Base Resolution	72	240	240	256
Phase Resolution (%)	100	100	100	100
Bandwidth (Hz/Px)	938	210	605	260
Orientation	Transversal	Sagittal	Sagittal	Transversal
N volumes	30 directions + 1 b0 x 10 averages	--	--	--
Other sequence-specific characteristics	b-value = 600 s/mm; reduced FOV, 15 slices	dual SPGR, two IR-SPGR images§	RF Phase Increment = 0, 180	#12 TEs, 5 ms increment, reduced FOV, 10 slices
Acquisition time	(10 x) 1 m 36 s	12 m 56 s	(4 x) 1m 42 s	13 m 51 s
**Number of subjects**				
N iPD	24	46	46	72
N PD-LRRK2	1	5	3	2
N PD-GBA	3	8	7	7

N RBD	0	15	15	20
N RBD-GBA	0	1	1	1
N aGBA	0	8	8	8
N HC	19	26	26	25
N total	47	109	106	135

Legend: iPD = idiopathic Parkinson's patients; PD-LRRK2 = Parkinson's patients with mutation of the LRRK2 gene; PD-GBA = Parkinson's patients with mutation of the GBA gene; RBD = patients with REM sleep behavior disorder (RBD); RBD-GBA = RBD patients with pathogenic mutation of the GBA gene; aLRRK2 = asymptomatic carriers of a pathogenic mutation of the LRRK2 gene; aGBA = asymptomatic carriers of a pathogenic mutation of the GBA gene; HC = Healthy controls. § Two IR-SPGR images to assess B1 field inhomogeneity (72 slices, 2.3 x1.1 x 2.2 mm^3, TE 3.7 ms, TR 8 ms, TI 350 ms and 450 ms).

Supplementary figures

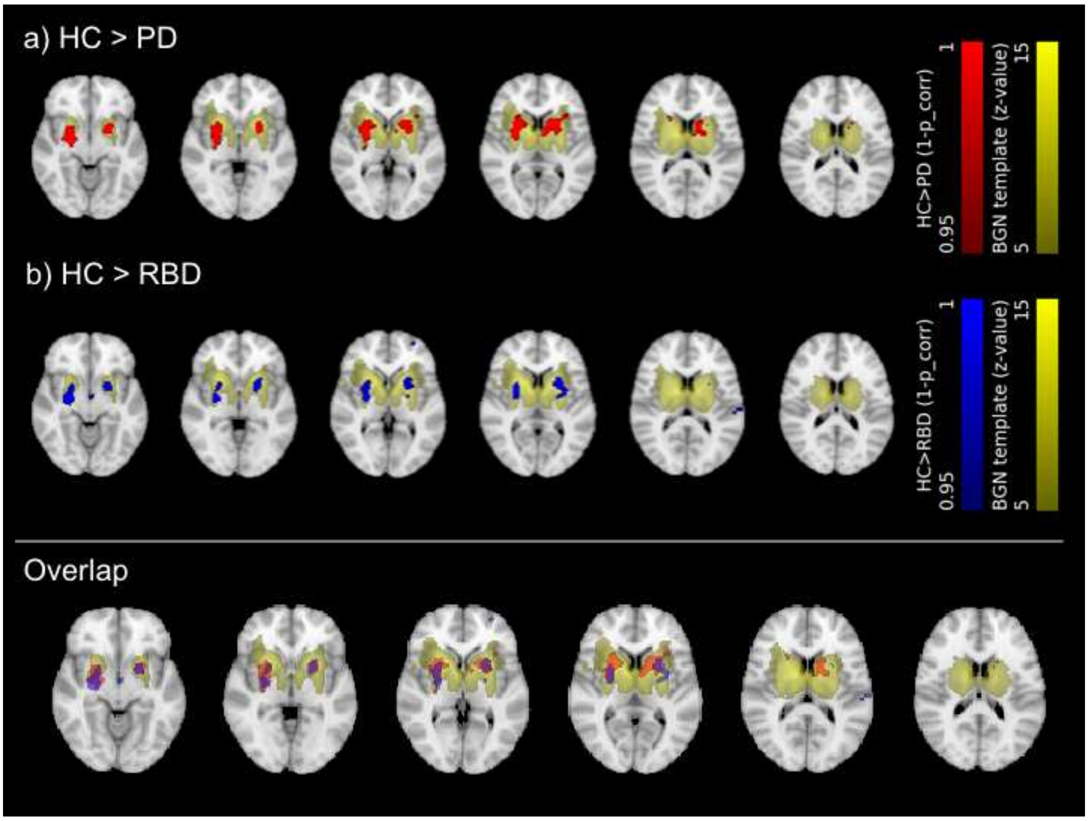

Figure S1. Resting state fMRI. Yellow underlay: basal ganglia network (BGN) template map, part of the template created with group ICA from 45 healthy controls external to the OPDC MRI sub-study (threshold of z=5 is applied for display purposes only; more details in [5], and

full template available online[6]). This was used in dual regression to extract subject-specific maps, which were then compared across groups. Results of the one-way ANOVA across 3 groups (n. PD = 103, n. RBD = 67, n. HC = 68) with post-hoc t-tests showed reduced functional connectivity in Parkinson's compared to controls (panel a, red) and reduced functional connectivity in RBD compared to controls (panel b, blue). Values displayed are 1-p>0.95, corresponding to p<0.05, corrected for multiple comparisons using threshold-free cluster enhancement. The bottom panel shows an overlap of the results. Full maps are available on NeuroVault (https://identifiers.org/neurovault.collection:5686). This is a replication of the analysis performed in [7] on a smaller sample (PD = 48, n. RBD = 26, n. HC = 23). These results are in line with the original study, although the effect size is smaller.

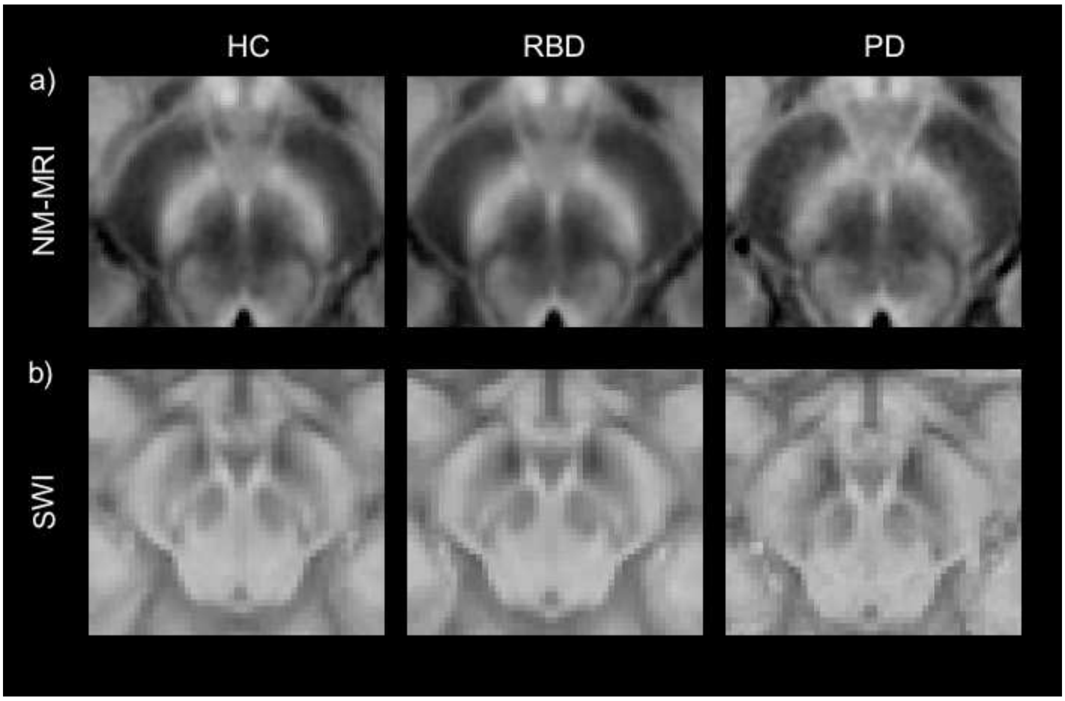

Figure S2. Novel sequences. In our cross-sectional sample, we observed a) progressive reduction of neuromelanin content in the substantia nigra pars compacta detected with NM-MRI (average maps from 25 HC, 42 RBD and 10 PD) and b) progressive reduction of the nigrosome-1 signal intensity (dorsal nigral hyperintensity - DNH - or 'swallow-tail sign') in SWI images (average maps from 32 HC, 44 RBD and 102 PD) from HC to RBD to manifest Parkinson's.

References

1. Vaillancourt DE, Spraker MB, Prodoehl J, et al. High-resolution diffusion tensor imaging in the substantia nigra of de novo Parkinson disease. *Neurology* 2009;72(16):1378-84. doi: 10.1212/01.wnl.0000340982.01727.6e
2. Deoni SC, Peters TM, Rutt BK. High-resolution T1 and T2 mapping of the brain in a clinically acceptable time with DESPOT1 and DESPOT2. *Magnetic resonance in medicine : official journal of the Society of Magnetic Resonance in Medicine / Society of Magnetic Resonance in Medicine* 2005;53(1):237-41. doi: 10.1002/mrm.20314
3. Baudrexel S, Nurnberger L, Rub U, et al. Quantitative mapping of T1 and T2* discloses nigral and brainstem pathology in early Parkinson's disease. *NeuroImage* 2010;51(2):512-20. doi: 10.1016/j.neuroimage.2010.03.005
4. Klein JC, Rolinski M, Griffanti L, et al. Cortical structural involvement and cognitive dysfunction in early Parkinson's disease. *NMR in biomedicine* 2018;31(4):e3900. doi: 10.1002/nbm.3900
5. Griffanti L, Rolinski M, Szewczyk-Krolikowski K, et al. Challenges in the reproducibility of clinical studies with resting state fMRI: An example in early Parkinson's disease. *NeuroImage* 2016;124(Pt A):704-13. doi: 10.1016/j.neuroimage.2015.09.021
6. OPDC MRI analysis [Available from: https://ora.ox.ac.uk/objects/uuid:8200af66-f438-4a7b-ad14-e8b032f0a9e7.
7. Rolinski M, Griffanti L, Piccini P, et al. Basal ganglia dysfunction in idiopathic REM sleep behaviour disorder parallels that in early Parkinson's disease. *Brain : a journal of neurology* 2016;139(Pt 8):2224-34. doi: 10.1093/brain/aww124
