# Supplementary material for: Cohort profile: the Oxford Parkinson’s Disease Centre Discovery Cohort MRI substudy (OPDC-MRI)
Source: BMJ Open. 2020 Aug 13;10(8):e034110. doi: 10.1136/bmjopen-2019-034110 (PMC7430482; doi:10.1136/bmjopen-2019-034110)
Supplement: Author's manuscript [file bmjopen-2019-034110.draft_revisions.pdf]

BMJ Open is committed to open peer review. As part of this commitment we make the peer review history of every article we publish publicly available.

When an article is published we post the peer reviewers' comments and the authors' responses online. We also post the versions of the paper that were used during peer review. These are the versions that the peer review comments apply to.

The versions of the paper that follow are the versions that were submitted during the peer review process. They are not the versions of record or the final published versions. They should not be cited or distributed as the published version of this manuscript.

BMJ Open is an open access journal and the full, final, typeset and author-corrected version of record of the manuscript is available on our site with no access controls, subscription charges or pay-per-view fees (http://bmjopen.bmj.com).

If you have any questions on BMJ Open's open peer review process please email info.bmjopen@bmj.com

BMJ Open

Cohort Profile: the Oxford Parkinson's Disease Centre Discovery Cohort Magnetic Resonance Imaging sub-study (OPDC-MRI)

Journal:	*BMJ Open*
Manuscript ID	bmjopen-2019-034110
Article Type:	Cohort profile
Date Submitted by the Author:	06-Sep-2019

Complete List of Authors:	Griffanti, Ludovica; University of Oxford, Wellcome Centre for Integrative Neuroimaging, Oxford Centre for Functional MRI of the Brain, Nuffield Department of Clinical Neurosciences<br>Klein, Johannes; University of Oxford, Wellcome Centre for Integrative Neuroimaging, Oxford Centre for Functional MRI of the Brain, Nuffield Department of Clinical Neurosciences; University of Oxford, Nuffield Department of Clinical Neurosciences<br>Szewczyk-Krolikowski, Konrad; University of Oxford, Nuffield Department of Clinical Neurosciences<br>Menke, Ricarda; University of Oxford, Wellcome Centre for Integrative Neuroimaging, Oxford Centre for Functional MRI of the Brain, Nuffield Department of Clinical Neurosciences<br>Rolinski, Michal; University of Oxford, Nuffield Department of Clinical Neurosciences; University of Bristol, Institute of Clinical Neurosciences<br>Barber, Thomas; University of Oxford, Nuffield Department of Clinical Neurosciences; University of Oxford, Wellcome Centre for Integrative Neuroimaging, Oxford Centre for Human Brain Activity, Department of Psychiatry<br>Lawton, Michael; University of Bristol, Population Health Sciences<br>Evetts, Samuel; University of Oxford, Nuffield Department of Clinical Neurosciences<br>Begeti, Faye; University of Oxford, Nuffield Department of Clinical Neurosciences<br>Crabbe, Marie; University of Oxford, Nuffield Department of Clinical Neurosciences<br>Rumbold, Jane; University of Oxford, Nuffield Department of Clinical Neurosciences<br>Wade-Martins, Richard; University of Oxford, Department of Physiology, Anatomy and Genetics<br>Hu, Michele; University of Oxford, Nuffield Department of Clinical Neurosciences<br>Mackay, Clare; University of Oxford, Wellcome Centre for Integrative Neuroimaging, Oxford Centre for Human Brain Activity, Department of Psychiatry; Oxford Health NHS Foundation Trust

Keywords:	Magnetic resonance imaging < RADIOLOGY & IMAGING, Cohort studies, Parkinson-s disease < NEUROLOGY, REM Sleep Behavior Disorder

I, the Submitting Author has the right to grant and does grant on behalf of all authors of the Work (as defined in the below author licence), an exclusive licence and/or a non-exclusive licence for contributions from authors who are: i) UK Crown employees; ii) where BMJ has agreed a CC-BY licence shall apply, and/or iii) in accordance with the terms applicable for US Federal Government officers or employees acting as part of their official duties; on a worldwide, perpetual, irrevocable, royalty-free basis to BMJ Publishing Group Ltd ("BMJ") its licensees and where the relevant Journal is co-owned by BMJ to the co-owners of the Journal, to publish the Work in this journal and any other BMJ products and to exploit all rights, as set out in our licence.

The Submitting Author accepts and understands that any supply made under these terms is made by BMJ to the Submitting Author unless you are acting as an employee on behalf of your employer or a postgraduate student of an affiliated institution which is paying any applicable article publishing charge ("APC") for Open Access articles. Where the Submitting Author wishes to make the Work available on an Open Access basis (and intends to pay the relevant APC), the terms of reuse of such Open Access shall be governed by a Creative Commons licence – details of these licences and which Creative Commons licence will apply to this Work are set out in our licence referred to above.

Other than as permitted in any relevant BMJ Author's Self Archiving Policies, I confirm this Work has not been accepted for publication elsewhere, is not being considered for publication elsewhere and does not duplicate material already published. I confirm all authors consent to publication of this Work and authorise the granting of this licence.

For peer review only

Cohort Profile: the Oxford Parkinson's Disease Centre Discovery Cohort Magnetic Resonance Imaging sub-study (OPDC-MRI)

Ludovica Griffanti[1,2], Johannes C Klein[1,2,3], Konrad Szewczyk-Krolikowski[1,3], Ricarda A L Menke[2], Michal Rolinski[1,3,4], Thomas R Barber[1,3,5], Michael Lawton[6], Samuel G Evetts[1,3], Faye Begeti[1,3], Marie Crabbe[1,3], Jane Rumbold[1,3], Richard Wade-Martins[1,7], Michele T. Hu[1,3], Clare E Mackay[1,5,8].

1 Oxford Parkinson's Disease Centre, Oxford, UK
2 Wellcome Centre for Integrative Neuroimaging, Oxford Centre for Functional MRI of the Brain, Nuffield Department of Clinical Neurosciences, University of Oxford, Oxford, UK
3 Nuffield Department of Clinical Neurosciences, University of Oxford, Oxford, UK
4 Institute of Clinical Neurosciences, University of Bristol, Bristol, UK
5 Wellcome Centre for Integrative Neuroimaging, Oxford Centre for Human Brain Activity, Department of Psychiatry, University of Oxford, Oxford, UK
6 Population Health Sciences, University of Bristol, Bristol, UK
7 Department of Physiology, Anatomy and Genetics, University of Oxford, Oxford, UK
8 Oxford Health NHS Foundation Trust, Oxford, UK

Number of tables: 3
Number of figures: 2
Abstract word count: 296
Manuscript word count (main text): 4278

Corresponding author: Professor Clare E. Mackay
E-mail: clare.mackay@ohba.ox.ac.uk
Address: Oxford Centre for Human Brain Activity (OHBA), University Department of Psychiatry, Warneford Hospital, Oxford, OX3 7JX
Telephone: +44 (0)1865 283802; Fax +44 (0)1865 793101

For peer review only

Abstract

Purpose: The Oxford Parkinson's Disease Centre (OPDC) Discovery Cohort magnetic resonance imaging (MRI) sub-study (OPDC-MRI) collects high quality multimodal brain MRI together with deep longitudinal clinical phenotyping in patients with Parkinson's, at-risk individuals and healthy elderly participants. The primary aim is to detect pathological changes in brain structure and function, and develop, together with the clinical data, biomarkers to stratify, predict and chart progression in early-stage Parkinson's and at-risk individuals.

Participants: Participants are recruited from the OPDC Discovery Cohort, a prospective, longitudinal study. Baseline MRI data is currently available for 290 participants: 119 patients with early idiopathic Parkinson's, 15 Parkinson's patients with pathogenic mutations of the LRRK2 or GBA genes, 68 healthy controls and 87 individuals at risk of Parkinson's (asymptomatic carriers of GBA mutation and patients with idiopathic rapid eye movement sleep behaviour disorder - RBD).

Findings to date: Differences in brain structure in early Parkinson's were found to be subtle, with small changes in the shape of the globus pallidus and evidence of alterations in microstructural integrity in the prefrontal cortex that correlated with performance on executive function tests. Brain function, as assayed with resting fMRI yielded more substantial differences, with basal ganglia connectivity reduced in early Parkinson's, and RBD, but not Alzheimer's, suggesting that the effect is pathology specific. Imaging of the substantia nigra with the more recent adoption of sequences sensitive to iron and neuromelanin content shows promising results in identifying early signs of Parkinsonian disease.

Future plans: Ongoing studies include the integration of multimodal MRI measures to improve discrimination power. Follow-up clinical data are now accumulating and will allow us to correlate baseline imaging measures to clinical disease progression. Follow-up MRI scanning started in 2015 and is currently ongoing, providing the opportunity for future longitudinal imaging analyses with parallel clinical phenotyping.

Keywords: Magnetic Resonance Imaging; Cohort Studies; Parkinson's Disease; REM Sleep Behavior Disorder

For peer review only - http://bmjopen.bmj.com/site/about/guidelines.xhtml

Article Summary

Strengths and limitations of this study

- High quality 3T MRI data in a very well phenotyped and longitudinally followed cohort of Parkinson's and RBD.
- All imaging data were acquired on the same MRI scanner, quite unique for a study of this duration. The protocol includes both standard sequences, comparable across other studies, and sequences acquired to investigate study-specific research questions.
- Clinical longitudinal data are acquired every 18 months and will be used to relate baseline imaging with clinical progression. Information about conversion to Parkinson's of the at-risk individuals will also be available, providing the ultimate validation of potential biomarkers. MRI follow-up is also ongoing, which will allow longitudinal imaging analyses.
- Statistical maps of published results and support data relative to the analyses are available to share.
- OPDC-MRI phenotyping is deep and relatively frequent, however the size of the cohort is not at the level of population-level cohort studies. MRI sequences are high quality, but could not exploit the latest advances in the field in order to maintain continuity.

For peer review only

Introduction

The Oxford Parkinson's Disease Centre (OPDC)[1] is a multidisciplinary research centre at the University of Oxford supported by Parkinson's UK with funds from The Monument Trust. It was established in 2010 and brings together world-leaders in clinical neurology, neuroepidemiology, neuroimaging, proteomics, genomics, molecular genetics, transgenic Parkinson's models, neuropharmacology, neurophysiology and neuropathology.

The centre was formed to understand the earliest events in the development of Parkinson's, ultimately with a view to identifying the changes that occur before motor symptoms become apparent.

The overarching goals of the OPDC are to:

- Understand the progression of Parkinson's
- Predict the onset of Parkinson's
- Identify potential drug targets for Parkinson's
- Develop new treatments that will prevent the development of Parkinson's in at-risk individuals.

To these aims, the research activity is structured around three overlapping themes: 1. improved clinical cohorts for development of novel biomarkers; 2. improved cellular and genetic models of Parkinson's pathologies and pathways; 3. novel animal models of early neuronal dysfunction in Parkinson's.

Within theme 1, the OPDC Discovery Cohort is one of the largest and best-characterised cohorts of people with early motor-manifest and prodromal Parkinson's in the world[2-4]. It is a prospective, longitudinal study that has recruited patients with early idiopathic Parkinson's, healthy controls and individuals at risk of Parkinson's.

The aim of the OPDC Discovery Cohort is to provide a wealth of data to better understand the biology of premotor and early Parkinson's, and to identify predictors of disease onset and progression. In addition to standardised assessments of motor and non-motor function, there is a particular interest in validating cutting-edge technologies to stratify, predict and chart progression in Parkinson's, including brain imaging, saccadometry, smart-phone and wearable assessments.

The subset of the OPDC Discovery Cohort that underwent brain magnetic resonance imaging (MRI) constitutes the OPDC Discovery MRI (OPDC-MRI) sub-study and is the focus of this paper. Driven by the emerging evidence of novel imaging markers

For peer review only - http://bmjopen.bmj.com/site/about/guidelines.xhtml

with high predictive value, like the study by Vaillancourt and colleagues[5] and others mentioned in [6], this project started with the collection of a small number of scans. Thanks to the promising results and to accumulating evidence in the field about the potential of MRI to serve as a biomarker for manifest (e.g.[7 8]) and pre-motor Parkinson's[9], the imaging sub-study was further expanded, with the aim to develop biomarkers derived from multimodal MRI in order to:

- Detect damage and changes in brain structure and function in early-stage Parkinson's and prodromal 'at-risk' individuals.
- Predict disease progression and understand its neural correlates.
- Stratify at-risk individuals to identify potential candidates for clinical trials.

In this cohort profile we will describe the cohort composition, the MRI data collected, and the processing pipelines that we developed. We will then report findings to date and illustrate our future plans for the cohort.

Cohort description

Eligibility criteria and recruitment

Participants of the OPDC-MRI sub-study were recruited from the OPDC Discovery cohort since 2010. Neurologists, Parkinson's nurses, geriatricians and GPs from participating hospitals in the Thames Valley area (total population 2.1 million) were asked to identify all idiopathic Parkinson's cases who were diagnosed by a neurologist (or a geriatrician with a specialist interest in Parkinson's) within the previous three years, according to the UK PD Society Brain Bank Criteria for clinically probable idiopathic Parkinson's disease[10]. All participating clinicians are regularly contacted to ensure screening of incident cases diagnosed since study onset. Eligible cases were approached by post and asked to contact the OPDC if interested in taking part in the study. Exclusion criteria for participation are: non-idiopathic parkinsonism, secondary parkinsonism due to head trauma or medication use, cognitive impairment precluding informed consent, dementia preceding motoric Parkinson's by one year suggestive of dementia with Lewy bodies, or other features of atypical parkinsonism syndromes such as multiple system atrophy, progressive supranuclear palsy, corticobasal degeneration.

More details on the recruitment process, assessment and exclusion criteria for the OPDC Discovery Cohort are described elsewhere[2 11]. For the MRI sub-study, we

For peer review only - http://bmjopen.bmj.com/site/about/guidelines.xhtml

aimed to scan Parkinson's patients as quickly as possible after enrolment (within 3 years of diagnosis), but due to logistics and patient unavailabilities, scanning was performed up to 6 years after diagnosis (see Table 1 for details). Parkinson's patients with more than mild head tremor or presence of dyskinesia/dystonia were excluded in order to minimise movement artefacts during imaging. The Parkinson's imaging cohort includes sporadic patients (iPD, N=119) and patients with known pathogenic mutations of the Leucine-rich repeat kinase 2 (LRRK2; G2019S and R1441C; N=5) or glucocerebrosidase (GBA; L444P and N370S; N=10) genes. Please see [4] for further details on GBA and LRRK2 genotyping methods.

The healthy control group is comprised of 68 participants with no family history of parkinsonism, many of whom were spouses and friends of Parkinson's participants. Healthy controls were not receiving any medications known to affect the dopaminergic system. The at-risk group includes 74 patients with idiopathic rapid eye movement (REM) sleep behavior disorder (RBD), diagnosed with polysomnography according to International Classification of Sleep Disorders criteria[12] (for more details see [4]), RBD patients with a pathogenic mutation of the GBA gene (N=3), and asymptomatic carriers of GBA gene pathogenic mutations (N=8).

Additional exclusion criteria for the MRI sub-study for all groups were contraindications to MRI scanning, including a history of claustrophobia, incompatible metal foreign body or suspicion of such, unresolved metallic injury to the eye, or inability to travel to Oxford without assistance.

The study was undertaken with the understanding and written consent of each subject, with the approval of the local NHS ethics committee, and in compliance with national legislation and the Declaration of Helsinki.

For peer review only

Data collection

Baseline MRI data were collected between November 2010 and December 2018. Follow-up data acquisition started in 2015 and is currently ongoing (see future plans section). Participants with Parkinson's were scanned in a clinically defined "off" state, a minimum of 12h after the withdrawal of their dopaminergic medications.

Clinical data

Participants receive extensive assessment in designated research clinics as part of their participation in the OPDC Discovery cohort. The assessment, performed by a nurse and neurologist, includes a structured general medical interview, detailed characterisation of motor and non-motor features, and cognitive assessment (see [2] for details). Patients are followed up clinically every 18 months, while controls only have a baseline clinic visit. In a research clinic, we cannot formally diagnose patients who convert from RBD to Parkinson’s (or another neurodegenerative disorder). However, where history and examination suggest conversion to neurodegenerative disease, we alert the treating clinicians who then establish the diagnosis and ensure clinical management is in place. On the day of scanning, an additional UPDRS III assessment was performed (“off” in Parkinson’s). Table 1 summarises the main demographic and clinical characteristics of the OPDC-MRI sub-study cohort.

-Table1-

Imaging data

Scanning was performed at the Oxford Centre for Clinical Magnetic Resonance Research (OCMR) using a 3T Siemens Trio MRI scanner (Siemens, Erlangen, Germany) equipped with a 12-channel receive-only head coil. The neuroimaging protocol includes both structural and functional sequences and lasts approximately 45-50 minutes.

Within the allocated time, five modalities were always acquired (*core sequences*), while the remaining time was used to experiment with novel sequences, which changed during the study. We report here the details of two of them, introduced at a later stage based on new information becoming available in the field, and which showed promising preliminary results. We describe the other additional sequences we experimented in the supplementary material.

For each modality, pipelines for preprocessing and analysis of MRI data were developed and applied to the data to extract single-subject imaging variables (both summary and voxel-wise measurements). Here below we describe for each modality the rationale for acquisition, as well as the main preprocessing steps performed on the images to derive the measures of interest.

The main acquisition parameters and the number of available scans per group for each of the *core sequences* are listed in Table 2.

For peer review only - http://bmjopen.bmj.com/site/about/guidelines.xhtml

-Table2-

T1-weighted MRI. The T1w MPRAGE (Fig. 1a-1d) offers very good contrast across tissue classes: grey matter (GM), white matter (WM), and cerebrospinal fluid (CSF). It is primarily used to study GM structural macroscopic tissue in both cortical and subcortical regions. In Parkinson's, cortical morphology in cognitively intact patients is generally reported to be normal or mildly altered, while impaired cognition and dementia in Parkinson's have been found to be associated with more severe patterns of cortical atrophy[6 13-15]. Non-motor symptoms have been also associated with structural changes in specific related brain networks (for a review see [6]). With this sequence we aimed to investigate potential differences in GM density across groups or relationships between GM and clinical variables, as well as taking into account the effect of structural changes when analysing functional MRI data.

The fsl_anat pipeline was used to perform brain-extraction, bias-field correction and 3-class (GM, WM, CSF) tissue-type segmentation with FAST[16]. The resulting images were then used to perform voxel-based morphometry analyses[17] and to generate voxel-wise confound regressors for resting state functional MRI statistical analyses. Subcortical structure segmentation was performed with FIRST[18], obtaining 3D meshes for each structure for each subject (used to perform vertex analysis), as well as volumetric images used to calculate volumes or as masks to extract average values from other modalities.

T1-weighted images with suppressed white matter signal (T1 WM nulled) show enhanced contrast across the basal ganglia structures (Fig.1e). This allows the detection of, for example, the boundary between the internal and external globus pallidus and a finer segmentation of this structure using MIST[19] (Fig.1f).

T2-weighted Fluid-attenuated inversion recovery (FLAIR). This sequence is commonly used to detect white matter abnormalities like leukoencephalopathies, demyelinating diseases and abnormalities of vascular origin. Regarding vascular pathology, it is used to detect white matter hyperintensities (WMH) (Fig. 1g,1h). With this sequence we wanted to characterise the amount and distribution of WMH in the cohort, look for differences across groups and assess the possible relationship with cardiovascular risk.

For peer review only - http://bmjopen.bmj.com/site/about/guidelines.xhtml

FLAIR images were brain extracted and bias field corrected using FAST[16]. WMHs were then automatically segmented on FLAIR images with BIANCA[20], a supervised segmentation tool which assigns to each voxel a probability of being a lesion, based on their intensity in FLAIR and T1 and their location (details and training dataset are online[21]). The probabilistic output was thresholded and restricted to the voxels located within a mask created from the T1-weighted scans (using the command make_bianca_mask), which excluded the cortex and subcortical structures. The total WMH volume was then calculated for each scan.

-Figure1-

Diffusion-weighted MRI (dMRI). Diffusion MRI is used to study the microstructure of brain WM *in vivo*. Also, it serves to virtually reconstruct putative WM tracts. The basis of dMRI is measurement of the random motion of water molecules (diffusion), which has a preferential orientation in the WM (i.e. is less restricted along than across the axons). It follows that the preferential direction of water diffusion is related to fibre orientation. The amount of diffusion directionality and restriction can inform about the microstructural environment of a voxel under study.

Diffusion-weighted images were acquired along 60 isotropically distributed diffusion directions (b-value of 1000 s/mm^2). Five additional images were acquired without diffusion weighting (b=0 s/mm^2). B0 inhomogeneity for diffusion imaging was measured using a dual-echo GRE sequence and the resulting phase and magnitude images were processed to produce field maps for correction of inhomogeneity-induced distortions.

Correction for b0-associated and eddy current-related distortion, as well as participant's movement, were performed using EDDY[22]. EDDY uses a generative probabilistic model to estimate inter- and intra-volume movements, displacements caused by field inhomogeneity, and distortions caused by eddy currents induced by the diffusion gradients. Additionally, automatic artefact rejection replaces slice drop-outs with model estimates[23 24]. The resultant 4D diffusion data were then fed into dtifit, which fits a diffusion tensor model at each voxel[25 26] and generates maps of tensor-derived measures to assess WM microstructural integrity: fractional anisotropy (FA), mean diffusivity (MD), axial diffusivity (AD) and radial diffusivity (RD). Finally, we ran bedpostX to produce fibre orientation estimates and their

For peer review only

respective uncertainties. This model can be used to perform probabilistic tractography for reconstructing WM pathways and assess their properties such as structural connectivity.

Resting-state functional MRI (rfMRI). rfMRI is used to investigate brain function without requiring the subject to undertake a specific task. Although potentially harder to interpret than task fMRI, rfMRI is not affected by subject's performance or compliance and allows to study different resting state networks, i.e. sets of brain regions sharing a common time-course of spontaneous fluctuations that have been associated with specific brain functions[27]. Among those, the basal ganglia network[28] (supplementary Figure S1) is of particular interest for the OPDC-MRI cohort.

In 28 iPD patients we also repeated the rfMRI sequence in the "on-state" 60–90 min after taking their own dopaminergic medication[11].

Firstly, images were motion corrected with MCFLIRT and the six rigid-body parameter time series extracted for each subject were used for subsequent cleaning. Mean relative displacement was also calculated to potentially exclude subjects with excessive motion and as possible confound metric in further analyses. Images were brain extracted, corrected for B0 inhomogeneities using field maps, spatially smoothed using a Gaussian kernel of FWHM of 6 mm, and temporally filtered using a high-pass filtering of 150 s. Single-subject probabilistic independent component analysis (ICA) was then performed with MELODIC[29] with automated dimensionality estimation, followed by automatic component classification with FSL-FIX[30 31] to identify and regress out the contribution of the artefactual components reflecting non-neuronal fluctuations (FIX training dataset available online[21]). The pre-processed functional data were registered to the individual's structural scan and standard space images using FLIRT and FNIRT[32 33], using boundary-based-registration. Single-subject resting state networks were derived with dual regression[34] and compared across subjects. The spatial maps used as spatial regressors in dual regression can be derived either from group-level ICA on the study-specific data[11] or using an external template (for more details regarding this choice see [35]). Supplementary Figure S1 shows the basal ganglia network map, part of the template used for the analyses. The full templates used for the studies published so far with data from the OPDC cohort are available online[21].

For peer review only - http://bmjopen.bmj.com/site/about/guidelines.xhtml

Regarding non-core sequences acquired in the OPDC-MRI sub-study, Table 3 shows the main acquisition parameters and the number of available subjects per group for two sequences introduced in 2016, which showed particularly promising results. In the Supplementary material we provide more information on the other sequences experimented in this study, which include quantitative T1 and T2 mapping, diffusion weighted imaging of the substantia nigra and multi-echo T2*-weighted images of the substantia nigra.

-Table3-

Neuromelanin-sensitive MRI (NM-MRI). The monaminergic neurons in the substantia nigra (SN) and locus coeruleus (LC) are rich in neuromelanin, a dark pigment that gives these structures their distinct colour. Neuromelanin is detectable with MRI as a hyperintense signal using modified T1-weighted sequences[36 37], which exploit the paramagnetic properties of the pigment, due to its iron content. According to pathological studies in Parkinson's[38], the SN and LC are affected early in the neurodegeneration process[39], making them an interesting target for the development of neuroimaging biomarkers. Studies using NM-MRI found a reduction of the hyperintense signal on NM-MRI in SN and/or LC in patients in patients with established Parkinson's[40 41] and RBD[42-44]. These promising findings drove the inclusion of this sequence in our protocol.

NM-MRI images acquired in OPDC-MRI were bias field corrected using FAST[16] and the transformations to the individual's structural scan (T1w) and standard space were calculated using FLIRT and FNIRT[32 33]. We also defined two reference regions of interest (ROIs) in MNI space (one for SN and one for LC) and used the average intensities within the ROI (registered in individual subject space) as normalization factors in subsequent analyses.

To also extract quantitative information on these images, we developed a segmentation method to automatically quantify the hyperintense signal in SN and LC (Figure 2a and 2b, preliminary results in [45]). The analysis on the whole sample is currently ongoing and support data related to the analyses (e.g. reference ROIs) will be available online[21].

For peer review only - http://bmjopen.bmj.com/site/about/guidelines.xhtml

T2-weighted images / susceptibility weighted imaging (SWI).* Susceptibility-weighted imaging (SWI) uses tissue magnetic susceptibility differences to enhance contrast in MRI. This is achieved by using the phase image in addition to the magnitude of T2*-weighted images. The phase image contains information about local susceptibility changes between tissues, which can be useful in measuring iron content. There are numerous neurologic disorders that can benefit from a sensitive method that monitors the amount of iron in the brain, whether in the form of deoxyhemoglobin, ferritin, or hemosiderin[46]. In Parkinson's, SWI has recently emerged as a promising sequence for evaluating the integrity of the substantia nigra[47]. In healthy subjects, the dorsolateral SN shows an area of signal hyperintensity, corresponding to nigrosome-I. This feature, described as a 'swallow tail' appearance[48], is lost in Parkinson's, since nigrosome-I is affected early by synuclein degeneration. Given the promising evidence for the 'swallow tail' to be a candidate biomarker for Parkinson's, we included SWI in our protocol.

The following pre-processing was applied to the T2* images to obtain the final SWI image: macroscopic phase artefacts removal was performed by high-pass filtering the phase images using a 50x50 FWHM window in Fourier space (window size selected empirically to suppress artefacts in the midbrain caused by nearby aerated structures). Then, paramagnetic phase components only were taken to the 4th power and multiplied with the magnitude images.

The presence/absence of the dorsal nigral hyperintensity, the 'swallow tail' sign, was visually rated for each subject (Figure 2c). We also calculated the transformations from the individual's SWI to structural scan (T1w) and standard space using FLIRT and FNIRT[32 33] to perform group comparisons. To extract quantitative information on these images, we also developed a method to automatically quantify the nigrosome-I hyperintense signal (Barber et al., under review [a]). The analysis on the whole sample is currently ongoing and support data related to the analyses will be available online[21].

---

[a] Barber TR; Griffanti, L.; Bradley, K. M.; McGowan, D. R.; Lo, C.; Mackay, C. E.; Hu, M. T.; Klein, J. C. Nigrosome 1 imaging in REM sleep behaviour disorder and its association with dopaminergic decline, (under review).

For peer review only - http://bmjopen.bmj.com/site/about/guidelines.xhtml

Patient and Public Involvement

The OPDC Discovery Cohort is designed by and for patients and is closely linked with the Parkinson's UK local support group. Patient representatives are also involved in the funding/renewal and strategic oversight processes, and sit on the Data Access Panel with casting votes. Results are disseminated to the study participants through annual newsletters, the OPDC website[1], and series of talks at participants' open days.

Findings to date

Regarding brain structure, we found no differences between early iPD patients and controls using relatively standard analyses of whole-brain GM volume and overall regional volumes. A subtle abnormality in the shape of the right pallidus was detected, and corresponded to differences in connecting WM pathways. However, the subtle nature of these changes makes it unlikely that morphometric analysis alone will be useful for early diagnosis of Parkinson's[49].

We found evidence of alterations in microstructural integrity in the prefrontal cortex that correlate with performance on executive function tests[50]. This was investigated using cortical measurements of macro- and microstructure and performing multi-modal linked ICA on structural, quantitative T1 and dMRI in early Parkinson's. Although these patients were cognitively intact, there were significant differences between Parkinson's and controls in a fronto-parietal component primarily driven by altered cortical diffusion (FA and MD). These were related to cognitive performance. Intriguingly, the frontal areas involved match the distribution of impairment in striatal dopaminergic projections reported previously. A study in an independent patient group found a similar association between frontal WM integrity and cognition, corroborating the idea of early frontal microstructural involvement[51].

Brain function, as assayed with resting fMRI yielded more substantial differences, with basal ganglia functional connectivity (BGFC) reduced in early iPD and increased upon administration of dopaminergic medication[11]. This alteration was not found in Alzheimer's[52], providing some confidence that the effect is pathology specific. A similar BGFC reduction to iPD was found in RBD[53] and was replicated subsequently, although with smaller effect size, in a larger sample of this cohort (https://identifiers.org/neurovault.collection:5686). To test whether a link between

For peer review only - http://bmjopen.bmj.com/site/about/guidelines.xhtml

BGFC and dopamine-related function was present in healthy aging, we conducted a multivariate analysis in a large population sample of healthy controls. We found an age-related and sex-dependent decline of connectivity, but no unique dopamine-related function seemed to have a link with BGFC beyond those detectable in and linearly correlated with healthy aging[54]. Our measure of BGFC was reproducible across different analysis settings[35]; however, these group differences have diminished with increasing sample size and have not been replicated on a different Parkinson's population so far (unpublished). At the single subject-level, the discriminatory power of BGFC increased when using a more sophisticated supervised learning algorithm[55], but further investigation is needed to assess the potential of rfMRI as a clinical biomarker.

Preliminary analyses of the novel sequences showed promising results. Measures of substantia nigra and locus coeruleus volumes extracted from NM-MRI were found to be reduced in RBD patients compared with controls (preliminary results in[45]). We also found a decrease in NM in the substantia nigra in Parkinson's with respect to controls, especially in Parkinson's with RBD, while the locus coeruleus seems more affected in RBD, in line with its role in REM sleep regulation.

Analysis of SWI images showed progressive reduction of the nigrosome-1 signal intensity (swallow-tail sign) from HC to RBD to manifest Parkinson's in our cross-sectional sample. Preliminary results in our RBD imaging cohort show that 28% of patients have pathological nigrosome imaging, defined as absence of the dorsal nigral hyperintensity that represents nigrosome-1. Intriguingly, these patients also have reduced dopamine transporter binding in the striatum, suggesting that nigral SWI may be able to identify individuals with dopaminergic decline. This MRI method may have the potential to enrich cohorts for future neuroprotective trials in RBD, where participants with a high likelihood of conversion to motor Parkinson's are sought to achieve clinical endpoints in a manageable timeframe (Barber et al., under review). However, longitudinal follow up to determine the true predictive value of SWI will be needed to confirm this.

Long-term follow-up

These baseline data already represent a rich source of data from a deeply phenotyped cohort. A key aspect of the OPDC-MRI cohort, however, is the longitudinal follow-up, which is ongoing. Clinical longitudinal data are acquired in the

For peer review only - http://bmjopen.bmj.com/site/about/guidelines.xhtml

Discovery cohort every 18 months, and therefore, we will be able to use them to relate baseline imaging with clinical progression. This will potentially allow us to stratify patients and at-risk individuals and predict their progression. Information about conversion to Parkinson's of at-risk individuals will also be available, providing the ultimate validation of potential biomarkers. In 2015 we also commenced the acquisition of longitudinal follow-up MRI after 5 years from baseline for Parkinson's and HC, and after 2.5-3 years for RBD. This difference in time after baseline was chosen because we predict that a number of the RBD patients will convert to Parkinson's, so we aim to collect multiple data points prior to conversion. In this way we hope to assess their trajectory during the prodromal phase and capture the associated brain changes.

Strengths and limitations of this study

The main strength of this cohort is the collection of high quality 3T MRI data in a very well-phenotyped longitudinal cohort of Parkinson's and at-risk individuals. The RBD dataset is one of the biggest brain MRI datasets available for this population of at-risk individuals.

Inevitably, a limitation is due to the trade-off between depth of the phenotyping and size of the cohort. OPDC-MRI phenotyping is deep and longitudinal, however the size of the cohort is relatively modest, particularly when compared with population-level cohort studies.

We are also aware of the inevitable selection bias for this cohort. We recruited people already participating in OPDC Discovery cohort and therefore our cohort reflects the demographic of the catchment area. Moreover, our participants are those who were already participating in a research study and declared they were happy to be contacted for imaging (self-selection). As a result, our cohort cannot fully capture the variability of Parkinson's and RBD patients (e.g. patients with severe motor dysfunction may be less able to travel and tolerate an MRI scan).

Another key strength of the OPDC-MRI cohort is the imaging protocol. All MRI data were acquired using the same MRI scanner, which is quite unique for a study of this duration. In this way all baseline data, as well as longitudinal MRI data will be truly comparable, with no effect due to scanner and/or protocol change. The protocol includes both standard sequences, which are also comparable with other studies, as

For peer review only

well as more experimental sequences acquired to investigate study-specific research questions.

We incorporated a trade-off between exploiting the latest techniques available in MRI and continuity of protocol throughout the study. While we could not use the most up-to-date sequences (e.g. our EPI images - dMRI and rfMRI - are not acquired with multiband acceleration), we managed to reach a balance by fixing the core sequences to maintain continuity, while changing the experimental sequences as the field evolved to include promising techniques as they became available.

As detailed in the previous section, the cohort will be enriched by collecting longitudinal follow-up MRI data. The challenge will be to be able to get data from those patients who have progressed quickly and will be in a more severe Parkinson's stage. While they may still be able to undergo a telephone interview or clinical assessment, they may not be willing or able to tolerate an MRI scan.

As described in more details in the "Collaboration and data sharing" section below, we have made statistical maps of our results publicly available, as well as support data relative to the analyses. The data presented here are also available to request (details below).

Collaboration and data sharing

Details about collaborating with OPDC can be found at https://www.opdc.ox.ac.uk/external-collaborations; OPDC is part of the CENTRE-PD twinning project (https://www.centre-pd.lu), we have ongoing international collaborations and are open for new proposals.

The data presented in this work (baseline imaging, demographics and clinical variables) will be available through the Dementias Platform UK (https://portal.dementiasplatform.uk), where data can be accessed by submitting a study proposal. Please note that longitudinal data will become available at a later stage.

Statistical maps are available on NeuroVault[56] for the following publications:

[11] https://neurovault.org/collections/2694/;

[52] https://identifiers.org/neurovault.collection:5448;

[53] (Results relative to a replication of the original study on an increased sample): https://identifiers.org/neurovault.collection:5686;

For peer review only

[35] https://identifiers.org/neurovault.collection:2953

[54] https://identifiers.org/neurovault.collection:2681.

Other types of support data related to the analyses are available online (https://ora.ox.ac.uk/objects/uuid:8200af66-f438-4a7b-ad14-e8b032f0a9e7)[21] and the repository will keep being populated as the analyses progress.

For peer review only

For peer review only - http://bmjopen.bmj.com/site/about/guidelines.xhtml

Tables

Table 1. Demographic and clinical characteristics of the OPDC-MRI cohort.

	iPD	PD-LRRK2	PD-GBA	RBD	RBD-GBA	aGBA	HC
N	119	5	10	74	3	8	68
Age [years] (mean±stdev)	64.1 ±10.1	66.0 ±11.6	63.8 ±10.3	65.8 ±7.6	61.2 ±7.4	65.9 ±7.6	65.9 ±8.7
Gender [M/F]	76/43	2/3	6/4	68/6	3/0	3/5	45/23
Parkinson's disease duration at time of MRI [years] (mean±stdev)	(n=117) 2.31 ±1.52	3.06 ±3.49	3.29 ±1.81	-	-	-	-
Time between MRI and closest clinical assessment [days] (mean±stdev)	(n=118) 108 ±104	79 ± 105	140 ±107	115 ± 90	6 ± 4	(n=6) 273 ±25	(n=64) 387±644
Levodopa equivalent dose (LEDD) (mean±stdev) §	(n=117) 335±243	340 ±198	463 ±243	-	-	-	-
Hoen & Yahr (mean±stdev) §	(n=118) 1.79±0.57	2.40 ±0.89	2.10 ±0.88	(n=71) 0.03 ±0.17	0	(n=6) 0	(n=64) 0
UPDRS III (mean±stdev) § #	(n=118) 24.0 ±10.4	41.2 ±17.8	28.4 ±14.9	(n=74) 4.5 ±4.0	3.0 ±1.7	(n=6) 3.3 ±4.2	(n=64) 1.8 ±2.5
MoCA (mean±stdev) § ##	(n=117) 26.4 ±2.7	26.0 ±2.2	24.4 ±3.1	(n=73) 25.6 ±2.7	(n=2) 26.0 ±0.0	(n=6) 26.7 ±1.9	(n=62) 27.5 ±2.0

When data is missing for some participants, the number of values available is specified in brackets.

§ = Evaluated at closest clinical assessment (controls only have a baseline clinic visit at enrolment).

# = Corrected for missing questions using the approach described in [57].

= Corrected for education.

Legend: iPD = idiopathic Parkinson's patients; PD-LRRK2 = Parkinson's patients with mutation of the LRRK2 gene; PD-GBA = Parkinson's patients with mutation of the GBA gene; RBD = patients with REM sleep behavior disorder (RBD); RBD-GBA = RBD patients with pathogenic mutation of the GBA gene; aLRRK2 = asymptomatic carriers of a pathogenic mutation of the LRRK2 gene; aGBA = asymptomatic carriers of a pathogenic mutation of the GBA gene; HC = healthy controls.

For peer review only - http://bmjopen.bmj.com/site/about/guidelines.xhtml

Table 2. MRI core-sequences: parameters used in the study and number of available datasets for each modality.

	T1	T1 WM nulled	T2-FLAIR	dMRI	rfMRI
Sequence type	3D, MPRAGE	3D, MPRAGE	2D, FLAIR	EPI	EPI
Period of acquisition	2010-2018 (ongoing)				
TR (ms)	2040	3000	9000	9300	2000
TE (ms)	4.7	3.4	90	94	28
TI (ms)	900	409	2500	--	--
Flip angle (degrees)	8	8	150	--	89
Voxel size (mm^3)	1x1x1	0.9x0.9x1	1.1x0.9x3	2x2x2	3x3x3.5
FoV read (mm)	192	240	220	192	192
Fov phase (%)	90.6	81.3	100	100	100
Base Resolution	192	256	256	96	64
Phase Resolution (%)	100	100	75	100	100
Bandwidth (Hz/Px)	130	130	201	1628	2368
Orientation	Transversal	Transversal	Transversal	Transversal	Transversal
N volumes	--	--	--	60 directions + 5 b=0	180
Other sequence-specific characteristics	--	--	--	b-value = 1000 s/mm^2; Echo spacing = 0.69 ms	Echo spacing = 0.49 ms; Eyes open
Acquisition time	5 m 56 s	9 m 8 s	5 m 8 s	11 m 11 s	6 m 4 s
**Number of subjects**					
N iPD	119	118	100	114	117
N PD-LRRK2	5	5	1	2	4
N PD-GBA	10	10	1	10	10
N RBD	74	74	68	74	74
N RBD-GBA	3	3	3	3	3
N aGBA	8	8	6	8	8
N HC	68	66	55	66	68
N total	287	284	234	277	284

Legend: iPD = idiopathic Parkinson's patients; PD-LRRK2 = Parkinson's patients with mutation of the LRRK2 gene; PD-GBA = Parkinson's patients with mutation of the GBA gene; RBD = patients with REM sleep behavior disorder (RBD); RBD-GBA = RBD patients with pathogenic mutation of the GBA gene; aLRRK2 = asymptomatic carriers of a pathogenic mutation of the LRRK2 gene; aGBA = asymptomatic carriers of a pathogenic mutation of the GBA gene; HC = healthy controls.

For peer review only

Table 3. MRI experimental sequences: parameters used in the study and number of available datasets for each modality. Other experimental sequences are listed in the Supplementary material.

	**Neuromelanin-sensitive (NM-MRI)**	**SWI**
Sequence type	2D, T1 with MTR[#]	3D, T2*-weighted
Period of acquisition	From 2016, ongoing	
TR (ms)	1400	27
TE (ms)	17	20
Flip angle (degrees)	180	15
Voxel size (mm)	0.8 x 0.8 x 2	0.9 x 0.9 x1.5
FoV read (mm)	200	220
Fov phase (%)	100	90.6
Base Resolution	256	256
Phase Resolution (%)	100	96
Bandwidth (Hz/Px)	257	120
Orientation	Transversal	Transversal
Other sequence-specific characteristics	reduced FOV[$]; GRAPPA (accel factor 2)	Whole brain, GRAPPA (accel factor 2)
Acquisition time	3 m 41 s	4 m 54 s
**Number of subjects**		
N iPD	28	30
N PD-LRRK2	1	1
N PD-GBA	0	0
N RBD	45	46
N RBD-GBA	1	1
N aGBA	0	0
N HC	31	37
N total	106	115

[#] sequence adapted from Schwarz et al., 2011 [40]; [$] 24 slices covering the substantia nigra and locus coeruleus. When needed, the number of slices was reduced to remain within the specific absorption rate (SAR) limits without altering other acquisition parameters. Legend: iPD = idiopathic Parkinson's patients; PD-LRRK2 = Parkinson's patients with mutation of the LRRK2 gene; PD-GBA = Parkinson's patients with mutation of the GBA gene; RBD = patients with REM sleep behavior disorder (RBD); RBD-GBA = RBD patients with pathogenic mutation of the GBA gene; aLRRK2 = asymptomatic carriers of a pathogenic mutation of the LRRK2 gene; aGBA = asymptomatic carriers of a pathogenic mutation of the GBA gene; HC = healthy controls.

For peer review only

Figure legends

Figure 1. Structural sequences and related processing. T1weighted MRI (a) is brain extracted and bias field corrected (b) to perform tissue-type segmentation (GM in red, WM in yellow, CSF in blue) (c) and subcortical structures segmentation (d). T1-weighted white matter nulled (e) allows better contrast in the subcortical structures, which allows, for example, the segmentation of the Globus Pallidus (zoom) into its internal (GPi, yellow) and external (GPe, light blue) portions (f). FLAIR images (g) are used to detect and quantify white matter hyperintensities (red) (h).

Figure 2. Novel sequences: Neuromelanin-sensitive (NM-MRI, left) and susceptibility weighted imaging (SWI, right). Examples of segmentation of (a) the substantia nigra and (b) the locus coeruleus as hyperintense areas on NM-MRI. (c) Examples of the presence (top) absence (bottom) of the ‘swallow tail’ sign on SWI.

For peer review only

Acknowledgments

The work was supported by the Monument Trust Discovery Award from Parkinson's UK (J-1403) and by the Wellcome Centre for Integrative Neuroimaging, the MRC Dementias Platform UK, the National Institute for Health Research (NIHR) Oxford Biomedical Research Centre (BRC), and the NIHR Oxford Health BRC (a partnership between Oxford Health NHS Foundation Trust and the University of Oxford). The views expressed are those of the authors and not necessarily those of the NHS, the NIHR or the Department of Health.

JCK acknowledges support from the NIHR Oxford Health Clinical Research Facility. MR received funding support from a NIHR Academic Clinical Lectureship and a NIHR Oxford BRC Doctoral Training Fellowship. TRB received funding support from a Wellcome Trust Doctoral Training Fellowship, and a Biomedical Research Council Career Development Fellowship. FB received funding support from National Institute for Health Research.

The authors would like to acknowledge all the participants and their families for participating in the study. They also thank Tim Quinnell, Oliver Bandmann, Gary Dennis, Zenobia Zaiwalla and Graham Lennox for patients recruitment and in-clinic data collection. They are thankful to the staff of the Oxford Centre for Magnetic Resonance (OCMR), in particular Jane Francis, Kathryn Lacey, Rebecca Mills and Steven Knight, and to Amandine Louvel and Katie Ahmed for administering the cohort.

Footnotes

Author Contributions

LG had a major role in data acquisition and analysis, interpreted the data and drafted the manuscript for intellectual content.

JCK had a major role in data acquisition and analysis, interpreted the data and contributed to major revisions of the manuscript for intellectual content.

KSK, RALM, MR and TRB had a major role in data acquisition and analysis, interpreted the data and revised the manuscript for intellectual content.

ML, SE, FB, had a major role in data analysis and revised the manuscript for intellectual content.

For peer review only

MC and JR had a major role in data acquisition and revised the manuscript for intellectual content.

RWM, MTU, and CEM designed and conceptualised the study, interpreted the data and revised the manuscript for intellectual content.

All authors reviewed, critically revised and approved the manuscript.

Conflicts of interest

Any conflict of interest is detailed in the authors' disclosure forms, included in the submission.

Data Sharing Statement

The data presented in this work will be available through the Dementias Platform UK (https://portal.dementiasplatform.uk), where data can be accessed by submitting a study proposal. Statistical maps are available on NeuroVault.org and support data related to the analyses are available online (https://ora.ox.ac.uk/objects/uuid:8200af66-f438-4a7b-ad14-e8b032f0a9e7).

For peer review only

References

1. Oxford Parkinson's Disease Centre (OPDC) [Available from: https://opdc.medsci.ox.ac.uk/home]

2. Szewczyk-Krolikowski K, Tomlinson P, Nithi K, et al. The influence of age and gender on motor and non-motor features of early Parkinson's disease: initial findings from the Oxford Parkinson Disease Center (OPDC) discovery cohort. *Parkinsonism Relat Disord* 2014;20(1):99-105. doi: 10.1016/j.parkreldis.2013.09.025

3. Lawton M, Baig F, Rolinski M, et al. Parkinson's Disease Subtypes in the Oxford Parkinson Disease Centre (OPDC) Discovery Cohort. *J Parkinsons Dis* 2015;5(2):269-79. doi: 10.3233/JPD-140523

4. Barber TR, Lawton M, Rolinski M, et al. Prodromal Parkinsonism and Neurodegenerative Risk Stratification in REM Sleep Behavior Disorder. *Sleep* 2017;40(8) doi: 10.1093/sleep/zsx071

5. Vaillancourt DE, Spraker MB, Prodoehl J, et al. High-resolution diffusion tensor imaging in the substantia nigra of de novo Parkinson disease. *Neurology* 2009;72(16):1378-84. doi: 10.1212/01.wnl.0000340982.01727.6e

6. Pyatigorskaya N, Gallea C, Garcia-Lorenzo D, et al. A review of the use of magnetic resonance imaging in Parkinson's disease. *Ther Adv Neurol Disord* 2014;7(4):206-20. doi: 10.1177/1756285613511507

7. Tuite P. Brain Magnetic Resonance Imaging (MRI) as a Potential Biomarker for Parkinson's Disease (PD). *Brain Sci* 2017;7(6) doi: 10.3390/brainsci7060068

8. Helmich RC, Vaillancourt DE, Brooks DJ. The Future of Brain Imaging in Parkinson's Disease. *J Parkinsons Dis* 2018;8(s1):S47-S51. doi: 10.3233/JPD-181482

9. Barber TR, Klein JC, Mackay CE, et al. Neuroimaging in pre-motor Parkinson's disease. *Neuroimage Clin* 2017;15:215-27. doi: 10.1016/j.nicl.2017.04.011

10. Hughes AJ, Daniel SE, Kilford L, et al. Accuracy of clinical diagnosis of idiopathic Parkinson's disease: a clinico-pathological study of 100 cases. *Journal of neurology, neurosurgery, and psychiatry* 1992;55(3):181-4.

11. Szewczyk-Krolikowski K, Menke RA, Rolinski M, et al. Functional connectivity in the basal ganglia network differentiates PD patients from controls. *Neurology* 2014;83(3):208-14. doi: 10.1212/wnl.0000000000000592 [published Online First: 2014/06/13]

12. Sateia MJ. International Classification of Sleep Disorders-Third Edition Highlights and Modifications. *Chest* 2014;146(5):1387-94. doi: 10.1378/chest.14-0970

13. Hanganu A, Monchi O. Structural Neuroimaging Markers of Cognitive Decline in Parkinson's Disease. *Parkinsons Dis* 2016;2016:3217960. doi: 10.1155/2016/3217960

14. Burton EJ, McKeith IG, Burn DJ, et al. Cerebral atrophy in Parkinson's disease with and without dementia: a comparison with Alzheimer's disease, dementia with Lewy bodies and controls. *Brain : a journal of neurology* 2004;127(Pt 4):791-800. doi: 10.1093/brain/awh088

15. Mak E, Su L, Williams GB, et al. Baseline and longitudinal grey matter changes in newly diagnosed Parkinson's disease: ICICLE-PD study. *Brain : a journal of neurology* 2015;138(Pt 10):2974-86. doi: 10.1093/brain/awv211

For peer review only

16. Zhang Y, Brady M, Smith S. Segmentation of brain MR images through a hidden Markov random field model and the expectation-maximization algorithm. *IEEE transactions on medical imaging* 2001;20(1):45-57. doi: 10.1109/42.906424 [published Online First: 2001/04/11]

17. Good CD, Johnsrude IS, Ashburner J, et al. A voxel-based morphometric study of ageing in 465 normal adult human brains. *NeuroImage* 2001;14(1 Pt 1):21-36. doi: 10.1006/nimg.2001.0786 [published Online First: 2001/08/30]

18. Patenaude B, Smith SM, Kennedy DN, et al. A Bayesian model of shape and appearance for subcortical brain segmentation. *NeuroImage* 2011;56(3):907-22. doi: 10.1016/j.neuroimage.2011.02.046

19. Visser E, Keuken MC, Douaud G, et al. Automatic segmentation of the striatum and globus pallidus using MIST: Multimodal Image Segmentation Tool. *NeuroImage* 2016;125:479-97. doi: 10.1016/j.neuroimage.2015.10.013

20. Griffanti L, Zamboni G, Khan A, et al. BIANCA (Brain Intensity AbNormality Classification Algorithm): A new tool for automated segmentation of white matter hyperintensities. *NeuroImage* 2016;141:191-205. doi: 10.1016/j.neuroimage.2016.07.018

21. OPDC MRI analysis [Available from: https://ora.ox.ac.uk/objects/uuid:8200af66-f438-4a7b-ad14-e8b032f0a9e7]

22. Andersson JLR, Sotiropoulos SN. An integrated approach to correction for off-resonance effects and subject movement in diffusion MR imaging. *NeuroImage* 2016;125:1063-78. doi: 10.1016/j.neuroimage.2015.10.019

23. Andersson JLR, Graham MS, Zsoldos E, et al. Incorporating outlier detection and replacement into a non-parametric framework for movement and distortion correction of diffusion MR images. *NeuroImage* 2016;141:556-72. doi: 10.1016/j.neuroimage.2016.06.058

24. Andersson JLR, Graham MS, Drobnjak I, et al. Towards a comprehensive framework for movement and distortion correction of diffusion MR images: Within volume movement. *NeuroImage* 2017;152:450-66. doi: 10.1016/j.neuroimage.2017.02.085

25. Basser PJ, Mattiello J, LeBihan D. Estimation of the effective self-diffusion tensor from the NMR spin echo. *J Magn Reson B* 1994;103(3):247-54.

26. Pierpaoli C, Jezzard P, Basser PJ, et al. Diffusion tensor MR imaging of the human brain. *Radiology* 1996;201(3):637-48. doi: 10.1148/radiology.201.3.8939209

27. Smith SM, Fox PT, Miller KL, et al. Correspondence of the brain's functional architecture during activation and rest. *Proceedings of the National Academy of Sciences of the United States of America* 2009;106(31):13040-5. doi: 10.1073/pnas.0905267106 [published Online First: 2009/07/22]

28. Robinson S, Basso G, Soldati N, et al. A resting state network in the motor control circuit of the basal ganglia. *BMC neuroscience* 2009;10:137. doi: 10.1186/1471-2202-10-137 [published Online First: 2009/11/26]

29. Beckmann CF, Smith SM. Probabilistic independent component analysis for functional magnetic resonance imaging. *IEEE transactions on medical imaging* 2004;23(2):137-52. doi: 10.1109/tmi.2003.822821 [published Online First: 2004/02/18]

For peer review only

30. Griffanti L, Salimi-Khorshidi G, Beckmann CF, et al. ICA-based artefact removal and accelerated fMRI acquisition for improved resting state network imaging. *NeuroImage* 2014;95:232-47. doi: 10.1016/j.neuroimage.2014.03.034 [published Online First: 2014/03/25]

31. Salimi-Khorshidi G, Douaud G, Beckmann CF, et al. Automatic denoising of functional MRI data: combining independent component analysis and hierarchical fusion of classifiers. *NeuroImage* 2014;90:449-68. doi: 10.1016/j.neuroimage.2013.11.046 [published Online First: 2014/01/07]

32. Jenkinson M, Smith S. A global optimisation method for robust affine registration of brain images. *Med Image Anal* 2001;5(2):143-56.

33. Andersson JL, Jenkinson M, Smith S. Non-linear registration aka Spatial normalisation FMRIB Technial Report TR07JA2. *FMRIB Analysis Group of the University of Oxford* 2007

34. Filippini N, MacIntosh BJ, Hough MG, et al. Distinct patterns of brain activity in young carriers of the APOE-epsilon4 allele. *Proceedings of the National Academy of Sciences of the United States of America* 2009;106(17):7209-14. doi: 10.1073/pnas.0811879106 [published Online First: 2009/04/10]

35. Griffanti L, Rolinski M, Szewczyk-Krolikowski K, et al. Challenges in the reproducibility of clinical studies with resting state fMRI: An example in early Parkinson's disease. *NeuroImage* 2016;124(Pt A):704-13. doi: 10.1016/j.neuroimage.2015.09.021

36. Sasaki M, Shibata E, Tohyama K, et al. Neuromelanin magnetic resonance imaging of locus ceruleus and substantia nigra in Parkinson's disease. *Neuroreport* 2006;17(11):1215-8. doi: 10.1097/01.wnr.0000227984.84927.a7

37. Chen X, Huddleston DE, Langley J, et al. Simultaneous imaging of locus coeruleus and substantia nigra with a quantitative neuromelanin MRI approach. *Magnetic resonance imaging* 2014;32(10):1301-6. doi: 10.1016/j.mri.2014.07.003

38. Braak H, Del Tredici K. Invited Article: Nervous system pathology in sporadic Parkinson disease. *Neurology* 2008;70(20):1916-25. doi: 10.1212/01.wnl.0000312279.49272.9f

39. Zucca FA, Segura-Aguilar J, Ferrari E, et al. Interactions of iron, dopamine and neuromelanin pathways in brain aging and Parkinson's disease. *Prog Neurobiol* 2017;155:96-119. doi: 10.1016/j.pneurobio.2015.09.012

40. Schwarz ST, Rittman T, Gontu V, et al. T1-weighted MRI shows stage-dependent substantia nigra signal loss in Parkinson's disease. *Mov Disord* 2011;26(9):1633-8. doi: 10.1002/mds.23722

41. Sulzer D, Cassidy C, Horga G, et al. Neuromelanin detection by magnetic resonance imaging (MRI) and its promise as a biomarker for Parkinson's disease. *NPJ Parkinsons Dis* 2018;4:11. doi: 10.1038/s41531-018-0047-3

42. Ehrminger M, Latimier A, Pyatigorskaya N, et al. The coeruleus/subcoeruleus complex in idiopathic rapid eye movement sleep behaviour disorder. *Brain : a journal of neurology* 2016;139(Pt 4):1180-8. doi: 10.1093/brain/aww006

43. Knudsen K, Fedorova TD, Hansen AK, et al. In-vivo staging of pathology in REM sleep behaviour disorder: a multimodality imaging case-control study. *The Lancet Neurology* 2018;17(7):618-28. doi: 10.1016/S1474-4422(18)30162-5

For peer review only - http://bmjopen.bmj.com/site/about/guidelines.xhtml

44. Pyatigorskaya N, Gaurav R, Arnaldi D, et al. Magnetic Resonance Imaging Biomarkers to Assess Substantia Nigra Damage in Idiopathic Rapid Eye Movement Sleep Behavior Disorder. *Sleep* 2017;40(11) doi: 10.1093/sleep/zsx149

45. Neuromelanin-MRI in REM sleep behaviour disorder predicts

dopaminergic dysfunction and clinical risk of Parkinson's disease - abstract n.176. International Parkinson and Movement Disorder Society; 2018. Movement Disorders.

46. Haacke EM, Mittal S, Wu Z, et al. Susceptibility-weighted imaging: technical aspects and clinical applications, part 1. *AJNR American journal of neuroradiology* 2009;30(1):19-30. doi: 10.3174/ajnr.A1400

47. Mahlknecht P, Krismer F, Poewe W, et al. Meta-analysis of dorsolateral nigral hyperintensity on magnetic resonance imaging as a marker for Parkinson's disease. *Mov Disord* 2017;32(4):619-23. doi: 10.1002/mds.26932

48. Schwarz ST, Afzal M, Morgan PS, et al. The 'swallow tail' appearance of the healthy nigrosome - a new accurate test of Parkinson's disease: a case-control and retrospective cross-sectional MRI study at 3T. *PloS one* 2014;9(4):e93814. doi: 10.1371/journal.pone.0093814

49. Menke RA, Szewczyk-Krolikowski K, Jbabdi S, et al. Comprehensive morphometry of subcortical grey matter structures in early-stage Parkinson's disease. *Human brain mapping* 2014;35(4):1681-90. doi: 10.1002/hbm.22282

50. Klein JC, Rolinski M, Griffanti L, et al. Cortical structural involvement and cognitive dysfunction in early Parkinson's disease. *NMR in biomedicine* 2018;31(4):e3900. doi: 10.1002/nbm.3900

51. Minett T, Su L, Mak E, et al. Longitudinal diffusion tensor imaging changes in early Parkinson's disease: ICICLE-PD study. *J Neurol* 2018;265(7):1528-39. doi: 10.1007/s00415-018-8873-0

52. Rolinski M, Griffanti L, Szewczyk-Krolikowski K, et al. Aberrant functional connectivity within the basal ganglia of patients with Parkinson's disease. *Neuroimage Clin* 2015;8:126-32. doi: 10.1016/j.nicl.2015.04.003

53. Rolinski M, Griffanti L, Piccini P, et al. Basal ganglia dysfunction in idiopathic REM sleep behaviour disorder parallels that in early Parkinson's disease. *Brain : a journal of neurology* 2016;139(Pt 8):2224-34. doi: 10.1093/brain/aww124

54. Griffanti L, Stratmann P, Rolinski M, et al. Exploring variability in basal ganglia connectivity with functional MRI in healthy aging. *Brain Imaging Behav* 2018;12(6):1822-27. doi: 10.1007/s11682-018-9824-1

55. Arya Z, Griffanti L, Mackay CE, et al. Iterative Dual LDA: A Novel Classification Algorithm for Resting State fMRI. *Lect Notes Comput Sc* 2016;10019:279-86. doi: 10.1007/978-3-319-47157-0_34

56. Gorgolewski KJ, Varoquaux G, Rivera G, et al. NeuroVault.org: a web-based repository for collecting and sharing unthresholded statistical maps of the human brain. *Front Neuroinform* 2015;9:8. doi: 10.3389/fninf.2015.00008

57. Goetz CG, Luo S, Wang L, et al. Handling missing values in the MDS-UPDRS. *Mov Disord* 2015;30(12):1632-8. doi: 10.1002/mds.26153

For peer review only - http://bmjopen.bmj.com/site/about/guidelines.xhtml

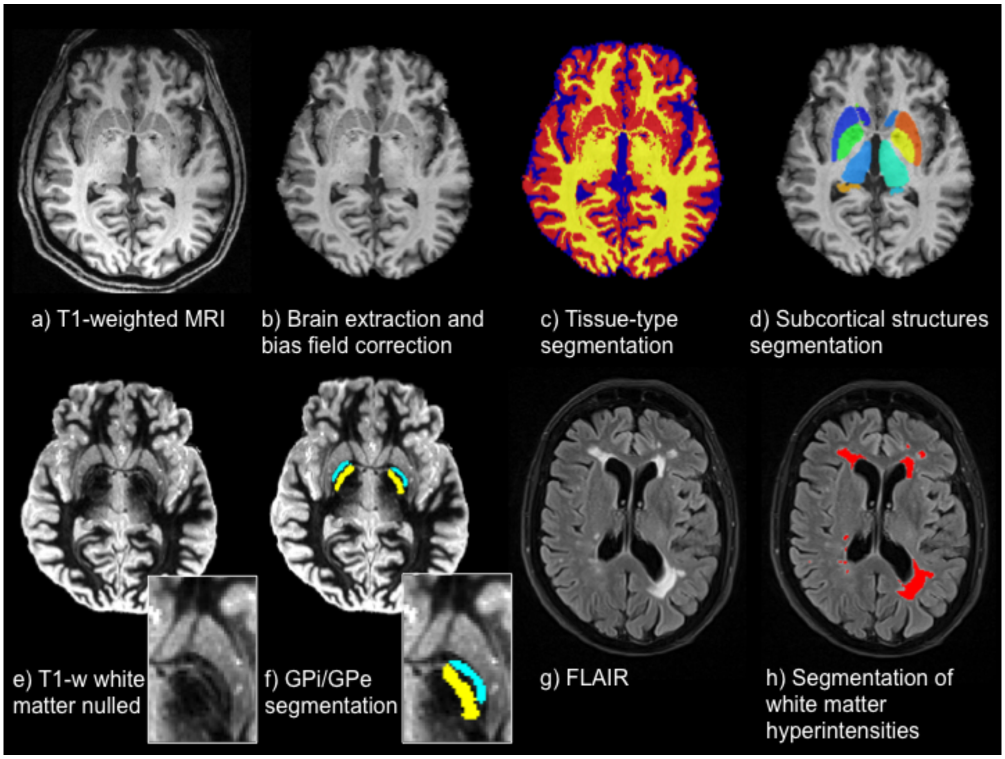

Structural sequences and related processing. T1weighted MRI (a) is brain extracted and bias field corrected (b) to perform tissue-type segmentation (GM in red, WM in yellow, CSF in blue) (c) and subcortical structures segmentation (d). T1-weighted white matter nulled (e) allows better contrast in the subcortical structures, which allows, for example, the segmentation of the Globus Pallidus (zoom) into its internal (GPi, yellow) and external (GPe, light blue) portions (f). FLAIR images (g) are used to detect and quantify white matter hyperintensities (red) (h).

254x190mm (72 x 72 DPI)

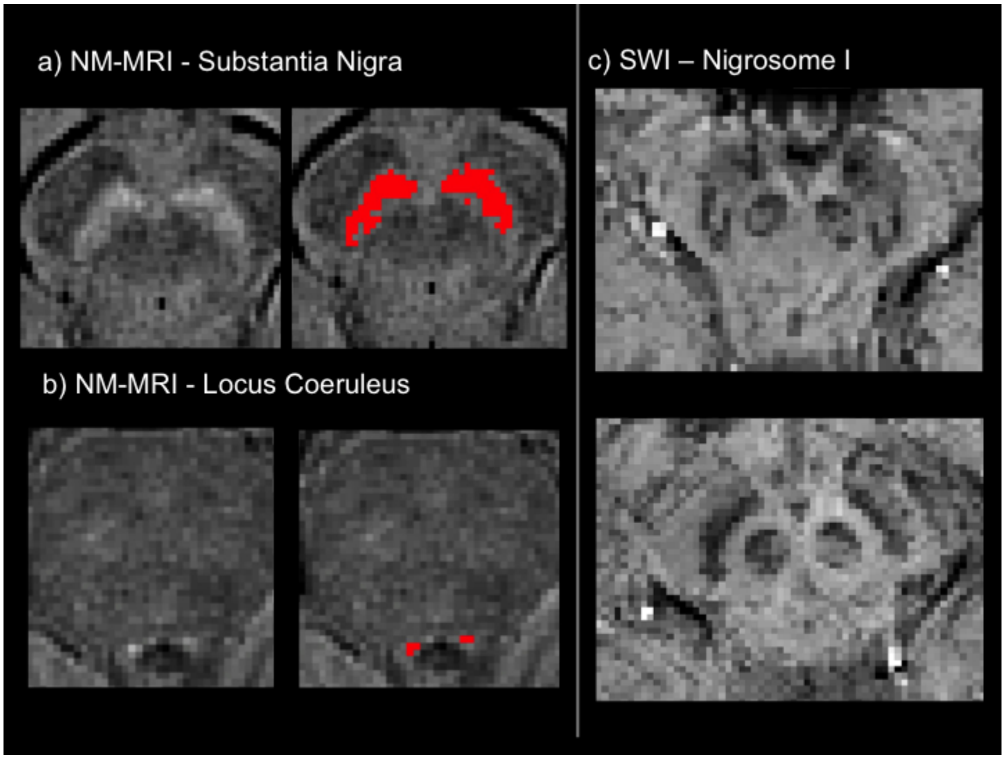

Novel sequences: Neuromelanin-sensitive (NM-MRI, left) and susceptibility weighted imaging (SWI, right). Examples of segmentation of (a) the substantia nigra and (b) the locus coeruleus as hyperintense areas on NM-MRI. (c) Examples of the presence (top) absence (bottom) of the 'swallow tail' sign on SWI.

254x190mm (72 x 72 DPI)

Supplementary material

Cohort Profile: the Oxford Parkinson's Disease Centre Discovery Cohort Magnetic Resonance Imaging sub-study (OPDC-MRI)

Griffanti et al.

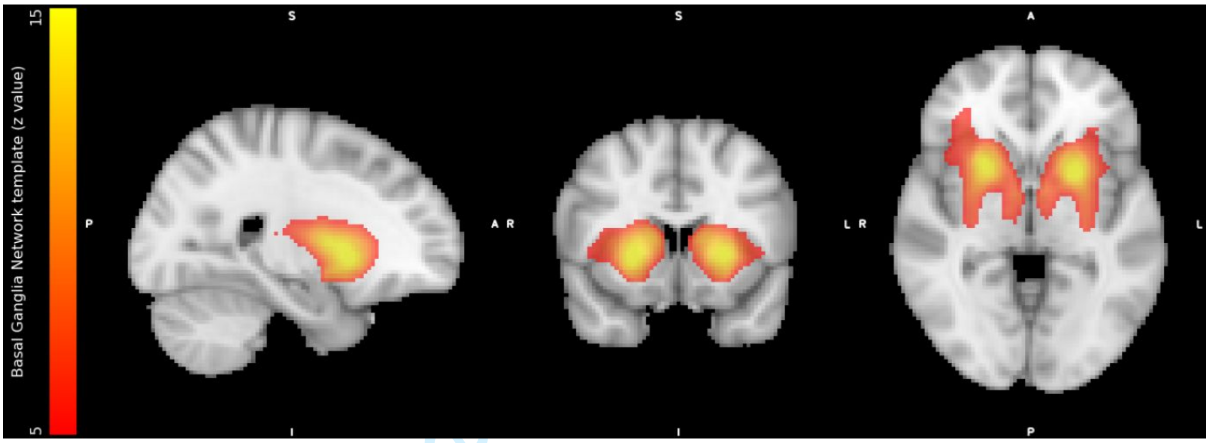

Figure S1. Resting state fMRI: basal ganglia network, part of the template used in dual regression to extract subject-specific maps. Full template available online[1]

Discontinued sequences

Supplementary Table S1 illustrates the main acquisition parameters and the number of available subjects per group for each of the sequences that were acquired between 2010 and 2016.

The diffusion-weighted sequence with reduced FOV centered on the substantia nigra (SN) was inspired by the study by Vaillancourt and colleagues [2], who found reduced FA in the SN of subjects with Parkinson's compared with controls, especially in the caudal compared with the rostral region of interest. We acquired a similar sequence in our first 50 participants, but did not find any difference between Parkinson's and controls (unpublished). We therefore decided to discontinue the sequence.

DESPOT1 and DESPOT2 (driven equilibrium single pulse observation of T1and T2, respectively) are a set of rapid combined T1 and T2 mapping approaches developed by Deoni and colleagues to be acquired in a clinically acceptable time [3]. They also demonstrated that by acquiring DESPOT2 in addition to DESPOT1 it is possible to retrospectively calculate 'synthetic' weighted images with arbitrary T1 or T2 weighting, since the underlying relaxation parameters are known. A study using quantitative MRI (qMRI) in early Parkinson's [4] found widespread reduction of midbrain T1 values contralateral to the clinically more severely affected limbs and

For peer review only

suggested that T1 may be a valuable marker for the monitoring of progressive neuronal loss in Parkinson's. T1 qMRI probes the chemical composition of the tissues, providing complementary information to the microstructural one available with diffusion imaging. In light of these findings we experimented with this sequence in OPDC-MRI. Quantitative T1 maps were generated using DESPOT-HiFi (dual SPGR, 144 slices) with two IR-SPGR images to assess B1 field inhomogeneity [3]. While we were able to use the T1 maps obtained from DESPOT1 [5], the quality of DESPOT2 obtained was not deemed sufficient to perform further analyses.

The study by Baudrexel and colleagues [4] also acquired multi-echo T2* and performed T2* mapping of the midbrain, which revealed a bilateral decrease of T2* values restricted to the SN, indicating a local increase in total iron content. They conclude that nigral T2* reductions might be associated with an increased general vulnerability for the development of the disorder. We therefore included this small FOV, multi ECHO T2* sequence to explore its diagnostic usefulness given that SN iron accumulation seems implicated in Parkinson's and T2* correlates with iron content.

This sequence was subsequently replaced with whole brain SWI because, despite not being able to provide T2* mapping (non multi-echo), it has a much shorter acquisition time and covers the whole brain, allowing, for example, to quantify iron content in the basal ganglia (see main text for details).

Table S1. Discontinued MRI experimental sequences: parameters used in the study and number of available datasets for each modality.

	**SN dMRI**	**qT1**	**qT2**	**SN GRE**
Sequence type	EPI	3D, DESPOT-HiFi	3D, DESPOT-HiFi	2D, T2*-weighted
Period of acquisition	2010-2013	2010-2014	2010-2014	2010-2016
TR (ms)	3000	8	55	648
TE (ms)	82	3.7	2.8	5 to 60[#]
TI (ms)	--	--	--	--
Flip angle (degrees)	90	4, 18	15, 60	45
Voxel size (mm)	0.8x0.8x4	1.1x1.1x1.1	1.1x1.1x1.1	1 x 1 x 2
FoV read (mm)	60	270	270	256
Fov phase (%)	41.7	83.3	83.3	100
Base Resolution	72	240	240	256
Phase Resolution (%)	100	100	100	100

For peer review only

Bandwidth (Hz/Px)	938	210	605	260
Orientation	Transversal	Sagittal	Sagittal	Transversal
N volumes	30 directions + 1 b0 x 10 averages (?)	--	--	--
Other sequence-specific characteristics	b-value = 600 s/mm; reduced FOV, 15 slices	dual SPGR, two IR-SPGR images§	RF Phase Increment = 0, 180	#12 TEs, 5 ms increment, reduced FOV, 10 slices
Acquisition time	(10 x) 1 m 36 s	12 m 56 s	(4 x) 1m 42 s	13 m 51 s
**Number of subjects**				
N iPD	24	46	46	72
N PD-LRRK2	1	5	3	2
N PD-GBA	3	8	7	7
N RBD	0	15	15	20
N RBD-GBA	0	1	1	1
N aGBA	0	8	8	8
N HC	19	26	26	25
N total	47	109	106	135

Legend: iPD = idiopathic Parkinson's patients; PD-LRRK2 = Parkinson's patients with mutation of the LRRK2 gene; PD-GBA = Parkinson's patients with mutation of the GBA gene; RBD = patients with REM sleep behavior disorder (RBD); RBD-GBA = RBD patients with pathogenic mutation of the GBA gene; aLRRK2 = asymptomatic carriers of a pathogenic mutation of the LRRK2 gene; aGBA = asymptomatic carriers of a pathogenic mutation of the GBA gene; HC = Healthy controls. § Two IR-SPGR images to assess B1 field inhomogeneity (72 slices, 2.3 x1.1 x 2.2 mm^3, TE 3.7 ms, TR 8 ms, TI 350 ms and 450 ms).

References

1. OPDC MRI analysis [https://ora.ox.ac.uk/objects/uuid:8200af66-f438-4a7b-ad14-e8b032f0a9e7].
2. Vaillancourt DE, Spraker MB, Prodoehl J, et al. High-resolution diffusion tensor imaging in the substantia nigra of de novo Parkinson disease. *Neurology* 2009;72(16):1378-84. doi: 10.1212/01.wnl.0000340982.01727.6e
3. Deoni SC, Peters TM, Rutt BK. High-resolution T1 and T2 mapping of the brain in a clinically acceptable time with DESPOT1 and DESPOT2. *Magnetic resonance in medicine : official journal of the Society of Magnetic Resonance in Medicine / Society of Magnetic Resonance in Medicine* 2005;53(1):237-41. doi: 10.1002/mrm.20314
4. Baudrexel S, Nurnberger L, Rub U, et al. Quantitative mapping of T1 and T2* discloses nigral and brainstem pathology in early Parkinson's disease. *NeuroImage* 2010;51(2):512-20. doi: 10.1016/j.neuroimage.2010.03.005

For peer review only

5. Klein JC, Rolinski M, Griffanti L, et al. Cortical structural involvement and cognitive dysfunction in early Parkinson's disease. *NMR in biomedicine* 2018;31(4):e3900. doi: 10.1002/nbm.3900

For peer review only

STROBE 2007 (v4) Statement—Checklist of items that should be included in reports of *cohort studies*

Section/Topic	Item #	Recommendation	Reported on page #
**Title and abstract**	1	(*a*) Indicate the study's design with a commonly used term in the title or the abstract	2
		(*b*) Provide in the abstract an informative and balanced summary of what was done and what was found	2
**Introduction**			
Background/rationale	2	Explain the scientific background and rationale for the investigation being reported	4
Objectives	3	State specific objectives, including any prespecified hypotheses	5
**Methods**			
Study design	4	Present key elements of study design early in the paper	5,6
Setting	5	Describe the setting, locations, and relevant dates, including periods of recruitment, exposure, follow-up, and data collection	6,7
Participants	6	(*a*) Give the eligibility criteria, and the sources and methods of selection of participants. Describe methods of follow-up	5-7
		(*b*) For matched studies, give matching criteria and number of exposed and unexposed	N/A
Variables	7	Clearly define all outcomes, exposures, predictors, potential confounders, and effect modifiers. Give diagnostic criteria, if applicable	5-7
Data sources/ measurement	8*	For each variable of interest, give sources of data and details of methods of assessment (measurement). Describe comparability of assessment methods if there is more than one group	7-12
Bias	9	Describe any efforts to address potential sources of bias	7-12
Study size	10	Explain how the study size was arrived at	5
Quantitative variables	11	Explain how quantitative variables were handled in the analyses. If applicable, describe which groupings were chosen and why	7-12
Statistical methods	12	(*a*) Describe all statistical methods, including those used to control for confounding	N/A
		(*b*) Describe any methods used to examine subgroups and interactions	N/A
		(*c*) Explain how missing data were addressed	N/A
		(*d*) If applicable, explain how loss to follow-up was addressed	N/A
		(*e*) Describe any sensitivity analyses	N/A
**Results**			

For peer review only

Participants	13*	(a) Report numbers of individuals at each stage of study—eg numbers potentially eligible, examined for eligibility, confirmed eligible, included in the study, completing follow-up, and analysed	Tables
		(b) Give reasons for non-participation at each stage	5,6
		(c) Consider use of a flow diagram	N/A
Descriptive data	14*	(a) Give characteristics of study participants (eg demographic, clinical, social) and information on exposures and potential confounders	Table 1
		(b) Indicate number of participants with missing data for each variable of interest	Table 2,3
		(c) Summarise follow-up time (eg, average and total amount)	N/A
Outcome data	15*	Report numbers of outcome events or summary measures over time	N/A
Main results	16	(*a*) Give unadjusted estimates and, if applicable, confounder-adjusted estimates and their precision (eg, 95% confidence interval). Make clear which confounders were adjusted for and why they were included	N/A
		(*b*) Report category boundaries when continuous variables were categorized	N/A
		(*c*) If relevant, consider translating estimates of relative risk into absolute risk for a meaningful time period	N/A
Other analyses	17	Report other analyses done—eg analyses of subgroups and interactions, and sensitivity analyses	N/A
**Discussion**			
Key results	18	Summarise key results with reference to study objectives	13
**Limitations**			
Interpretation	20	Give a cautious overall interpretation of results considering objectives, limitations, multiplicity of analyses, results from similar studies, and other relevant evidence	13-15
Generalisability	21	Discuss the generalisability (external validity) of the study results	13-15
**Other information**			
Funding	22	Give the source of funding and the role of the funders for the present study and, if applicable, for the original study on which the present article is based	21

*Give information separately for cases and controls in case-control studies and, if applicable, for exposed and unexposed groups in cohort and cross-sectional studies.

Note: An Explanation and Elaboration article discusses each checklist item and gives methodological background and published examples of transparent reporting. The STROBE checklist is best used in conjunction with this article (freely available on the Web sites of PLoS Medicine at http://www.plosmedicine.org/, Annals of Internal Medicine at http://www.annals.org/, and Epidemiology at http://www.epidem.com/). Information on the STROBE Initiative is available at www.strobe-statement.org.

For peer review only

BMJ Open

Cohort Profile: the Oxford Parkinson's Disease Centre Discovery Cohort Magnetic Resonance Imaging sub-study (OPDC-MRI)

Journal:	*BMJ Open*
Manuscript ID	bmjopen-2019-034110.R1
Article Type:	Cohort profile
Date Submitted by the Author:	21-Feb-2020

Complete List of Authors:	Griffanti, Ludovica; University of Oxford, Wellcome Centre for Integrative Neuroimaging, Oxford Centre for Functional MRI of the Brain, Nuffield Department of Clinical Neurosciences<br>Klein, Johannes; University of Oxford, Wellcome Centre for Integrative Neuroimaging, Oxford Centre for Functional MRI of the Brain, Nuffield Department of Clinical Neurosciences; University of Oxford, Nuffield Department of Clinical Neurosciences<br>Szewczyk-Krolikowski, Konrad; University of Oxford, Nuffield Department of Clinical Neurosciences<br>Menke, Ricarda; University of Oxford, Wellcome Centre for Integrative Neuroimaging, Oxford Centre for Functional MRI of the Brain, Nuffield Department of Clinical Neurosciences<br>Rolinski, Michal; University of Oxford, Nuffield Department of Clinical Neurosciences; University of Bristol, Institute of Clinical Neurosciences<br>Barber, Thomas; University of Oxford, Nuffield Department of Clinical Neurosciences; University of Oxford, Wellcome Centre for Integrative Neuroimaging, Oxford Centre for Human Brain Activity, Department of Psychiatry<br>Lawton, Michael; University of Bristol, Population Health Sciences<br>Evetts, Samuel; University of Oxford, Nuffield Department of Clinical Neurosciences<br>Begeti, Faye; University of Oxford, Nuffield Department of Clinical Neurosciences<br>Crabbe, Marie; University of Oxford, Nuffield Department of Clinical Neurosciences<br>Rumbold, Jane; University of Oxford, Nuffield Department of Clinical Neurosciences<br>Wade-Martins, Richard; University of Oxford, Department of Physiology, Anatomy and Genetics<br>Hu, Michele; University of Oxford, Nuffield Department of Clinical Neurosciences<br>Mackay, Clare; University of Oxford, Wellcome Centre for Integrative Neuroimaging, Oxford Centre for Human Brain Activity, Department of Psychiatry; Oxford Health NHS Foundation Trust

**Primary Subject Heading**:	Neurology
Secondary Subject Heading:	Radiology and imaging

Keywords:	Magnetic resonance imaging < RADIOLOGY & IMAGING, Cohort studies, Parkinson-s disease < NEUROLOGY, REM Sleep Behavior Disorder

SCHOLARONE™
Manuscripts

I, the Submitting Author has the right to grant and does grant on behalf of all authors of the Work (as defined in the below author licence), an exclusive licence and/or a non-exclusive licence for contributions from authors who are: i) UK Crown employees; ii) where BMJ has agreed a CC-BY licence shall apply, and/or iii) in accordance with the terms applicable for US Federal Government officers or employees acting as part of their official duties; on a worldwide, perpetual, irrevocable, royalty-free basis to BMJ Publishing Group Ltd ("BMJ") its licensees and where the relevant Journal is co-owned by BMJ to the co-owners of the Journal, to publish the Work in this journal and any other BMJ products and to exploit all rights, as set out in our licence.

The Submitting Author accepts and understands that any supply made under these terms is made by BMJ to the Submitting Author unless you are acting as an employee on behalf of your employer or a postgraduate student of an affiliated institution which is paying any applicable article publishing charge ("APC") for Open Access articles. Where the Submitting Author wishes to make the Work available on an Open Access basis (and intends to pay the relevant APC), the terms of reuse of such Open Access shall be governed by a Creative Commons licence – details of these licences and which Creative Commons licence will apply to this Work are set out in our licence referred to above.

Other than as permitted in any relevant BMJ Author's Self Archiving Policies, I confirm this Work has not been accepted for publication elsewhere, is not being considered for publication elsewhere and does not duplicate material already published. I confirm all authors consent to publication of this Work and authorise the granting of this licence.

For peer review only

Cohort Profile: the Oxford Parkinson's Disease Centre Discovery Cohort Magnetic Resonance Imaging sub-study (OPDC-MRI)

Ludovica Griffanti[1,2], Johannes C Klein[1,2,3], Konrad Szewczyk-Krolikowski[1,3], Ricarda A L Menke[2], Michal Rolinski[1,3,4], Thomas R Barber[1,3,5], Michael Lawton[6], Samuel G Evetts[1,3], Faye Begeti[1,3], Marie Crabbe[1,3], Jane Rumbold[1,3], Richard Wade-Martins[1,7], Michele T. Hu[1,3], Clare E Mackay[1,5,8].

1 Oxford Parkinson's Disease Centre, Oxford, UK
2 Wellcome Centre for Integrative Neuroimaging, Oxford Centre for Functional MRI of the Brain, Nuffield Department of Clinical Neurosciences, University of Oxford, Oxford, UK
3 Nuffield Department of Clinical Neurosciences, University of Oxford, Oxford, UK
4 Institute of Clinical Neurosciences, University of Bristol, Bristol, UK
5 Wellcome Centre for Integrative Neuroimaging, Oxford Centre for Human Brain Activity, Department of Psychiatry, University of Oxford, Oxford, UK
6 Population Health Sciences, University of Bristol, Bristol, UK
7 Department of Physiology, Anatomy and Genetics, University of Oxford, Oxford, UK
8 Oxford Health NHS Foundation Trust, Oxford, UK

Number of tables: 3
Number of figures: 2
Abstract word count: 286
Manuscript word count (main text): 4691

Corresponding author: Professor Clare E. Mackay
E-mail: clare.mackay@ohba.ox.ac.uk
Address: Oxford Centre for Human Brain Activity (OHBA), University Department of Psychiatry, Warneford Hospital, Oxford, OX3 7JX
Telephone: +44 (0)1865 283802; Fax +44 (0)1865 793101

For peer review only

Abstract

***Purpose**:* The Oxford Parkinson's Disease Centre (OPDC) Discovery Cohort magnetic resonance imaging (MRI) sub-study (OPDC-MRI) collects high quality multimodal brain MRI together with deep longitudinal clinical phenotyping in patients with Parkinson's, at-risk individuals and healthy elderly participants. The primary aim is to detect pathological changes in brain structure and function, and develop, together with the clinical data, biomarkers to stratify, predict and chart progression in early-stage Parkinson's and at-risk individuals.

Participants: Participants are recruited from the OPDC Discovery Cohort, a prospective, longitudinal study. Baseline MRI data is currently available for 290 participants: 119 patients with early idiopathic Parkinson's, 15 Parkinson's patients with pathogenic mutations of the LRRK2 or GBA genes, 68 healthy controls and 87 individuals at risk of Parkinson's (asymptomatic carriers of GBA mutation and patients with idiopathic rapid eye movement sleep behaviour disorder - RBD).

Findings to date: Differences in brain structure in early Parkinson's were found to be subtle, with small changes in the shape of the globus pallidus and evidence of alterations in microstructural integrity in the prefrontal cortex that correlated with performance on executive function tests. Brain function, as assayed with resting fMRI yielded more substantial differences, with basal ganglia connectivity reduced in early Parkinson's, and RBD. Imaging of the substantia nigra with the more recent adoption of sequences sensitive to iron and neuromelanin content shows promising results in identifying early signs of Parkinsonian disease.

Future plans: Ongoing studies include the integration of multimodal MRI measures to improve discrimination power. Follow-up clinical data are now accumulating and will allow us to correlate baseline imaging measures to clinical disease progression. Follow-up MRI scanning started in 2015 and is currently ongoing, providing the opportunity for future longitudinal imaging analyses with parallel clinical phenotyping.

Keywords: Magnetic Resonance Imaging; Cohort Studies; Parkinson's Disease; REM Sleep Behavior Disorder

For peer review only

Article Summary

Strengths and limitations of this study

- High quality 3T MRI data in a very well phenotyped and longitudinally followed cohort of Parkinson’s and RBD were acquired on the same MRI scanner, quite unique for a study of this duration.
- Clinical longitudinal data are acquired every 18 months, information about conversion to Parkinson’s of the at-risk individuals will be available, and MRI follow-up is ongoing.
- Statistical maps of published results and support data relative to the analyses are available to share.
- OPDC-MRI phenotyping is deep and relatively frequent, however the size of the cohort is not at the level of population-level cohort studies.
- MRI sequences are high quality, but could not exploit the latest advances in the field in order to maintain continuity.

For peer review only

Introduction

The Oxford Parkinson's Disease Centre (OPDC)[1] is a multidisciplinary research centre at the University of Oxford supported by Parkinson's UK with funds from The Monument Trust. It was established in 2010 and brings together world-leaders in clinical neurology, neuroepidemiology, neuroimaging, proteomics, genomics, molecular genetics, transgenic Parkinson's models, neuropharmacology, neurophysiology and neuropathology.

The centre was formed to understand the earliest events in the development of Parkinson's, ultimately with a view to identifying the changes that occur before motor symptoms become apparent.

The overarching goals of the OPDC are to:

- Understand the progression of Parkinson's
- Predict the onset of Parkinson's
- Identify potential drug targets for Parkinson's
- Develop new treatments that will prevent the development of Parkinson's in at-risk individuals.

To these aims, the research activity is structured around three overlapping themes: 1. improved clinical cohorts for development of novel biomarkers; 2. improved cellular and genetic models of Parkinson's pathologies and pathways; 3. novel animal models of early neuronal dysfunction in Parkinson's.

Within theme 1, the OPDC Discovery Cohort is one of the largest and best-characterised cohorts of people with early motor-manifest and prodromal Parkinson's in the world[2-4]. It is a prospective, longitudinal study that has recruited patients with early idiopathic Parkinson's, healthy controls and individuals at risk of Parkinson's.

The aim of the OPDC Discovery Cohort is to provide a wealth of data to better understand the biology of premotor and early Parkinson's, and to identify predictors of disease onset and progression. In addition to standardised assessments of motor and non-motor function, there is a particular interest in validating cutting-edge technologies to stratify, predict and chart progression in Parkinson's, including brain imaging, saccadometry, smart-phone and wearable assessments.

The subset of the OPDC Discovery Cohort that underwent brain magnetic resonance imaging (MRI) constitutes the OPDC Discovery MRI (OPDC-MRI) sub-study and is the focus of this paper. Driven by the emerging evidence of novel imaging markers

For peer review only - http://bmjopen.bmj.com/site/about/guidelines.xhtml

with high predictive value, like the study by Vaillancourt and colleagues[5] and others mentioned in[6], this project started with the collection of a small number of scans. Thanks to the promising results and to accumulating evidence in the field about the potential of MRI to serve as a biomarker for manifest (e.g.[7 8]) and pre-motor Parkinson's[9], the imaging sub-study was further expanded, with the aim to develop biomarkers derived from multimodal MRI in order to:

- Detect damage and changes in brain structure and function in early-stage Parkinson's and prodromal 'at-risk' individuals.
- Predict disease progression and understand its neural correlates.
- Stratify at-risk individuals to identify potential candidates for clinical trials.

In this cohort profile we will describe the cohort composition, the MRI data collected, and the processing pipelines that we developed. We will then report findings to date and illustrate our future plans for the cohort.

Cohort description

Eligibility criteria and recruitment

Participants of the OPDC-MRI sub-study were recruited from the OPDC Discovery cohort since 2010. For the main study (OPDC Discovery), neurologists, Parkinson's nurses, geriatricians and GPs from participating hospitals in the Thames Valley area (total population 2.1 million) were asked to identify all idiopathic Parkinson's cases who were diagnosed by a neurologist (or a geriatrician with a specialist interest in Parkinson's) within the previous three years, according to the UK PD Society Brain Bank Criteria for clinically probable idiopathic Parkinson's disease[10]. Self-selection bias is unavoidable in this scenario. Furthermore, the study group reflects the demographics of the Thames valley area. All participating clinicians are regularly contacted to ensure screening of incident cases diagnosed since study onset. Eligible cases were approached by post and asked to contact the OPDC if interested in taking part in the study. Patients were assessed in research clinics and their diagnosis was further confirmed by a neurologist specialising in movement disorders. Patients were excluded if confidence in the diagnosis was below 90% at that point. Exclusion criteria for participation assessed by a neurologist specialising in movement disorders, are: non-idiopathic parkinsonism, secondary parkinsonism due to head trauma or medication use, cognitive impairment precluding informed

For peer review only

For peer review only - http://bmjopen.bmj.com/site/about/guidelines.xhtml

consent, dementia preceding motoric Parkinson's by one year suggestive of dementia with Lewy bodies, or other features of atypical parkinsonism syndromes such as multiple system atrophy, progressive supranuclear palsy, corticobasal degeneration.

More details on the recruitment process, assessment and exclusion criteria for the OPDC Discovery Cohort are described elsewhere[2 11]. For the MRI sub-study, we aimed to scan Parkinson's patients as quickly as possible after enrolment (within 3 years of diagnosis), but due to logistics and patient unavailabilities, scanning was performed up to 6 years after diagnosis (see Table 1 for details). Parkinson's patients with more than mild head tremor or presence of dyskinesia/dystonia were excluded since movements artefacts would be likely be too severe to obtain usable images.

Genetic testing for known pathogenic mutation of the glucocerebrosidase gene (GBA; L444P and N370S) and Leucine-rich repeat kinase 2 gene (LRRK2; G2019S and R1441C) was performed on all participants in the OPDC Discovery Cohort who consented to it. In the OPDC-MRI cohort the results are currently available for 82% of the participants for the LRRK2 screening and for 96% of the participants for the GBA screening (two participants did not give consent). The details of the genetic testing procedure are available in [4].

The Parkinson's imaging cohort includes 119 sporadic patients (iPD), 5 patients with pathogenic mutations of the LRRK2 gene (PD-LRRK2) and 10 patients with pathogenic mutation of the GBA gene (PD-GBA).

The healthy control (HC) group is comprised of 68 participants also part of OPDC Discovery. Many of them were spouses and friends of Parkinson's participants with no first- or second-degree relatives diagnosed with Parkinson's. Healthy controls were not receiving any medications known to affect the dopaminergic system and absence of Parkinson's diagnosis was confirmed by a neurologist specialising in movement disorder. The at-risk group includes 74 patients with idiopathic rapid eye movement (REM) sleep behavior disorder (RBD), diagnosed with polysomnography according to International Classification of Sleep Disorders criteria[12] (for more details see[4]), RBD patients with a pathogenic mutation of the GBA gene (RBD-GBA, N=3), and asymptomatic carriers of GBA gene pathogenic mutations (aGBA, N=8).

Additional exclusion criteria for the MRI sub-study for all groups were contraindications to MRI scanning, including a history of claustrophobia,

For peer review only

incompatible metal foreign body or suspicion of such, unresolved metallic injury to the eye, or inability to travel to Oxford without assistance.

The study was undertaken with the understanding and written consent of each subject, with the approval of the local NHS ethics committee (National Research Ethics Service (NRES) Committee South Central – Oxford A (Ref:16/SC/0108) and Oxford C (Ref:15/SC/0117); Berkshire Research Ethics Committee (Ref: 10/H0505/71)), and in compliance with national legislation and the Declaration of Helsinki.

For peer review only

Data collection

Baseline MRI data were collected between November 2010 and December 2018. Follow-up data acquisition started in 2015 and is currently ongoing (see future plans section). Participants with Parkinson's were scanned in a clinically defined "off" state, a minimum of 12h after the withdrawal of their dopaminergic medications.

Clinical data

Participants receive extensive assessment in designated research clinics as part of their participation in the OPDC Discovery cohort. The assessment, performed by a nurse and neurologist, includes a structured general medical interview, detailed characterisation of motor and non-motor features, and cognitive assessment (see Supplementary table S1 and [2 13] for details). Patients are followed up clinically every 18 months, while controls only have a baseline clinic visit. In a research clinic, we cannot formally diagnose patients who convert from RBD to Parkinson's (or another neurodegenerative disorder). However, where history and examination suggest conversion to neurodegenerative disease, we alert the treating clinicians who then establish the diagnosis and ensure clinical management is in place. On the day of scanning, an additional UPDRS III assessment was performed ("off" in Parkinson's). Table 1 summarises the main demographic and clinical characteristics of the OPDC-MRI sub-study cohort.

-Table1-

Imaging data

For peer review only - http://bmjopen.bmj.com/site/about/guidelines.xhtml

Scanning was performed at the Oxford Centre for Clinical Magnetic Resonance Research (OCMR) using a 3T Siemens Trio MRI scanner (Siemens, Erlangen, Germany) equipped with a 12-channel receive-only head coil and foam cushions were used to minimize head motion. The neuroimaging protocol includes both structural and functional sequences and lasts approximately 45-50 minutes.

Within the allocated time, five modalities were always acquired (*core sequences*), while the remaining time was used to experiment with novel sequences, which changed during the study. We report here the details of two of them, introduced at a later stage based on new information becoming available in the field, and which showed promising preliminary results. We describe the other additional sequences we experimented in the supplementary material.

For each modality, pipelines for pre-processing and analysis of MRI data were developed and applied to the data to extract single-subject imaging variables (both summary and voxel-wise measurements). Here below we describe for each modality the rationale for acquisition, as well as the main pre-processing steps performed on the images to derive the measures of interest.

The main acquisition parameters and the number of available scans per group for each of the *core sequences* are listed in Table 2.

-Table2-

T1-weighted MRI. The T1w MPRAGE (Fig. 1a-1d) offers very good contrast across tissue classes: grey matter (GM), white matter (WM), and cerebrospinal fluid (CSF). It is primarily used to study GM structural macroscopic tissue in both cortical and subcortical regions. In Parkinson's, cortical morphology in cognitively intact patients is generally reported to be normal or mildly altered, while impaired cognition and dementia in Parkinson's have been found to be associated with more severe patterns of cortical atrophy[6 14-16]. Non-motor symptoms have been also associated with structural changes in specific related brain networks (for a review see[6]). With this sequence we aimed to investigate potential differences in GM density across groups or relationships between GM and clinical variables, as well as taking into account the effect of structural changes when analysing functional MRI data.

The fsl_anat pipeline was used to perform brain-extraction, bias-field correction and 3-class (GM, WM, CSF) tissue-type segmentation with FAST[17]. The resulting images

For peer review only - http://bmjopen.bmj.com/site/about/guidelines.xhtml

were then used to perform voxel-based morphometry analyses[18] and to generate voxel-wise confound regressors for resting state functional MRI statistical analyses. Subcortical structure segmentation was performed with FIRST[19], obtaining 3D meshes for each structure for each subject (used to perform vertex analysis), as well as volumetric images used to calculate volumes or as masks to extract average values from other modalities.

T1-weighted images with suppressed white matter signal (T1 WM nulled) show enhanced contrast across the basal ganglia structures (Fig.1e). This allows the detection of, for example, the boundary between the internal and external globus pallidus and a finer segmentation of this structure using MIST[20] (Fig.1f).

T2-weighted Fluid-attenuated inversion recovery (FLAIR). This sequence is commonly used to detect white matter abnormalities like leukoencephalopathies, demyelinating diseases and abnormalities of vascular origin. Regarding vascular pathology, it is used to detect white matter hyperintensities (WMH) (Fig. 1g,1h). With this sequence we wanted to characterise the amount and distribution of WMH in the cohort, look for differences across groups and assess the possible relationship with cardiovascular risk.

FLAIR images were brain extracted and bias field corrected using FAST[17]. WMHs were then automatically segmented on FLAIR images with BIANCA[21], a supervised segmentation tool which assigns to each voxel a probability of being a lesion, based on their intensity in FLAIR and T1 and their location (details and training dataset are online[22]). The probabilistic output was thresholded and restricted to the voxels located within a mask created from the T1-weighted scans (using the command make_bianca_mask), which excluded the cortex and subcortical structures. The total WMH volume was then calculated for each scan.

-Figure1-

Diffusion-weighted MRI (dMRI). Diffusion MRI is used to study the microstructure of brain WM *in vivo*. Also, it serves to virtually reconstruct putative WM tracts. The basis of dMRI is measurement of the random motion of water molecules (diffusion), which has a preferential orientation in the WM (i.e. is less restricted along than across the axons). It follows that the preferential direction of water diffusion is related

For peer review only - http://bmjopen.bmj.com/site/about/guidelines.xhtml

to fibre orientation. The amount of diffusion directionality and restriction can inform about the microstructural environment of a voxel under study.

Diffusion-weighted images were acquired along 60 isotropically distributed diffusion directions (b-value of 1000 s/mm^2). Five additional images were acquired without diffusion weighting (b=0 s/mm^2). B0 inhomogeneity for diffusion imaging was measured using a dual-echo GRE sequence and the resulting phase and magnitude images were processed to produce field maps for correction of inhomogeneity-induced distortions.

Correction for b0-associated and eddy current-related distortion, as well as participant's movement, were performed using EDDY[23]. EDDY uses a generative probabilistic model to estimate inter- and intra-volume movements, displacements caused by field inhomogeneity, and distortions caused by eddy currents induced by the diffusion gradients. Additionally, automatic artefact rejection replaces slice drop-outs with model estimates[24 25]. The resultant 4D diffusion data were then fed into dtifit, which fits a diffusion tensor model at each voxel[26 27] and generates maps of tensor-derived measures to assess WM microstructural integrity: fractional anisotropy (FA), mean diffusivity (MD), axial diffusivity (AD) and radial diffusivity (RD). Finally, we ran bedpostX to produce fibre orientation estimates and their respective uncertainties. This model can be used to perform probabilistic tractography for reconstructing WM pathways and assess their properties such as structural connectivity.

Resting-state functional MRI (rfMRI). rfMRI is used to investigate brain function without requiring the subject to undertake a specific task. Although potentially harder to interpret than task fMRI, rfMRI is not affected by subject's performance or compliance and allows to study different resting state networks, i.e. sets of brain regions sharing a common time-course of spontaneous fluctuations that have been associated with specific brain functions[28]. Among those, the basal ganglia network[29] (shown in yellow in supplementary Figure S1) is of particular interest for the OPDC-MRI cohort. rfMRI data were acquired with eyes open. In 28 iPD patients we also repeated the rfMRI sequence in the "on" state 60–90 min after taking their own dopaminergic medication[11].

Firstly, images were motion corrected with MCFLIRT and the six rigid-body parameter time series extracted for each subject were used for subsequent cleaning.

For peer review only - http://bmjopen.bmj.com/site/about/guidelines.xhtml

Mean relative displacement was also calculated to potentially exclude subjects with excessive motion and as possible confound metric in further analyses (e.g. to verify that there was no significant difference in mean displacement across groups[30 31]). Images were brain extracted, corrected for B0 inhomogeneities using field maps, spatially smoothed using a Gaussian kernel of FWHM of 6 mm, and temporally filtered using a high-pass filtering of 150 s. Single-subject probabilistic independent component analysis (ICA) was then performed with MELODIC[32] with automated dimensionality estimation, followed by automatic component classification with FSL-FIX[33 34] to identify the contribution of the artefactual components reflecting non-neuronal fluctuations (FIX training dataset available online[22]). The contributions of the motion parameter time series and of artefactual components were regressed out from the data. The pre-processed functional data were registered to the individual's structural scan and standard space images using FLIRT and FNIRT[35 36], using boundary-based-registration. Single-subject resting state networks were derived with dual regression[37] and compared across subjects. The spatial maps used as spatial regressors in dual regression can be derived either from group-level ICA on the study-specific data[11] or using an external template (for more details regarding this choice see [38]). Supplementary Figure S1 shows the basal ganglia network map, part of the template used for the analyses. The full templates used for the studies published so far with data from the OPDC cohort are available online[22].

Regarding non-core sequences acquired in the OPDC-MRI sub-study, Table 3 shows the main acquisition parameters and the number of available subjects per group for two sequences introduced in 2016, which showed particularly promising results. In the Supplementary material and table S2 we provide more information on the other sequences experimented in this study, which include quantitative T1 and T2 mapping, diffusion weighted imaging of the substantia nigra and multi-echo T2*-weighted images of the substantia nigra.

-Table3-

Neuromelanin-sensitive MRI (NM-MRI). The monaminergic neurons in the substantia nigra (SN) and locus coeruleus (LC) are rich in neuromelanin, a dark pigment that gives these structures their distinct colour. Neuromelanin is detectable with MRI as a

For peer review only

hyperintense signal using modified T1-weighted sequences[39 40], which exploit the paramagnetic properties of the pigment, due to its iron content. According to pathological studies in Parkinson's[41], the SN and LC are affected early in the neurodegeneration process[42], making them an interesting target for the development of neuroimaging biomarkers. Studies using NM-MRI found a reduction of the hyperintense signal on NM-MRI in SN and/or LC in patients in patients with established Parkinson's[43 44] and RBD[45-47]. These promising findings drove the inclusion of this sequence in our protocol.

NM-MRI images acquired in OPDC-MRI were bias field corrected using FAST[17] and the transformations to the individual's structural scan (T1w) and standard space were calculated using FLIRT and FNIRT[35 36]. We also defined two reference regions of interest (ROIs) in MNI space (one for SN and one for LC) and used the average intensities within the ROI (registered in individual subject space) as normalization factors in subsequent analyses.

To also extract quantitative information on these images, we developed a segmentation method to automatically quantify the hyperintense signal in SN and LC (Figure 2a and 2b, preliminary results in [48]). The analysis on the whole sample is currently ongoing and support data related to the analyses (e.g. reference ROIs) will be available online[22].

T2-weighted images / susceptibility weighted imaging (SWI).* Susceptibility-weighted imaging (SWI) uses tissue magnetic susceptibility differences to enhance contrast in MRI. This is achieved by using the phase image in addition to the magnitude of T2*-weighted images. The phase image contains information about local susceptibility changes between tissues, which can be useful in measuring iron content. There are numerous neurologic disorders that can benefit from a sensitive method that monitors the amount of iron in the brain, whether in the form of deoxyhemoglobin, ferritin, or hemosiderin[49]. In Parkinson's, SWI has recently emerged as a promising sequence for evaluating the integrity of the substantia nigra[50]. In healthy subjects, the dorsolateral SN shows an area of signal hyperintensity, corresponding to nigrosome-I. This feature, described as a 'swallow tail' appearance[51], is lost in Parkinson's, since nigrosome-I is affected early by synuclein degeneration. Given the promising evidence for the 'swallow tail' to be a candidate biomarker for Parkinson's, we included SWI in our protocol.

For peer review only - http://bmjopen.bmj.com/site/about/guidelines.xhtml

The following pre-processing was applied to the T2* images to obtain the final SWI image: macroscopic phase artefacts removal was performed by high-pass filtering the phase images using a 50x50 FWHM window in Fourier space (window size selected empirically to suppress artefacts in the midbrain caused by nearby aerated structures). Then, paramagnetic phase components only were taken to the 4th power and multiplied with the magnitude images.

The presence/absence of the dorsal nigral hyperintensity, the 'swallow tail' sign, was visually rated for each subject (Figure 2c). We also calculated the transformations from the individual's SWI to structural scan (T1w) and standard space using FLIRT and FNIRT[35 36] to perform group comparisons. To extract quantitative information on these images, we also developed a method to automatically quantify the nigrosome-I hyperintense signal[52]. Support data related to the analyses will be available online[22].

-Figure2-

Patient and Public Involvement

The OPDC Discovery Cohort is designed by and for patients and is closely linked with the Parkinson's UK local support group. Patient representatives are also involved in the funding/renewal and strategic oversight processes, and sit on the Data Access Panel with casting votes. Results are disseminated to the study participants through annual newsletters, the OPDC website[1], and series of talks at participants' open days.

Findings to date

Regarding brain structure, we found no differences between early iPD patients and controls using relatively standard analyses of whole-brain GM volume and overall regional volumes. A subtle abnormality in the shape of the right pallidus was detected, and corresponded to differences in connecting WM pathways. However, the subtle nature of these changes makes it unlikely that morphometric analysis alone will be useful for early diagnosis of Parkinson's[53].

We found evidence of alterations in microstructural integrity in the prefrontal cortex that correlate with performance on cognitive tests[54]. This was investigated using cortical measurements of macro- and microstructure and performing multi-modal

For peer review only - http://bmjopen.bmj.com/site/about/guidelines.xhtml

linked ICA on structural, quantitative T1 and dMRI in early Parkinson's. Although these patients were cognitively intact, there were significant differences between Parkinson's and controls in a fronto-parietal component primarily driven by altered cortical diffusion (FA and MD). These were related to cognitive performance (Montreal Cognitive Assessment – MoCA). Intriguingly, the frontal areas involved match the distribution of impairment in striatal dopaminergic projections reported previously. A study in an independent patient group found a similar association between frontal WM integrity and cognition, corroborating the idea of early frontal microstructural involvement[55].

Brain function, as assayed with resting fMRI yielded more substantial differences, with basal ganglia functional connectivity (BGFC) reduced in early iPD and increased upon administration of dopaminergic medication[11]. When comparing HC and iPD from the OPDC-MRI sub-study with a group of patients with Alzheimer's acquired with the same scanner and protocol[56], no BGNFC alteration was found in Alzheimer's[30], providing some confidence that the effect is pathology specific. A similar BGFC reduction to iPD was found in RBD[31] and was replicated subsequently, although with smaller effect size, in a larger sample of this cohort (https://identifiers.org/neurovault.collection:5686). We did not find significant association between BGFC and motor performance (UPDRS-III)[11 30] also when taking laterality into account[31]. To test whether a link between BGFC and dopamine-related function was present in healthy aging, we conducted a multivariate analysis in a large population sample of healthy controls. We found an age-related and sex-dependent decline of connectivity, but no unique dopamine-related function seemed to have a link with BGFC beyond those detectable in and linearly correlated with healthy aging[57]. Our measure of BGFC was reproducible across different analysis settings[38]; however, these group differences have diminished with increasing sample size (supplementary Figure S2) and have not been replicated on a different Parkinson's population so far. At the single subject-level, the discriminatory power of BGFC increased when using a more sophisticated supervised learning algorithm[58], but further investigation is needed to assess the potential of rfMRI as a clinical biomarker.

Measures of substantia nigra and locus coeruleus volumes extracted from NM-MRI were found to be reduced in RBD patients compared with controls (preliminary results in[48]). We also found a decrease in NM in the substantia nigra in Parkinson's

For peer review only - http://bmjopen.bmj.com/site/about/guidelines.xhtml

with respect to controls, especially in Parkinson's with RBD, while the locus coeruleus seems more affected in RBD, in line with its role in REM sleep regulation.

SWI images showed progressive reduction of the nigrosome-1 signal intensity (dorsal nigral hyperintensity - DNH - or 'swallow-tail sign') from HC to RBD to manifest Parkinson's in our cross-sectional sample (supplementary Figure S2). In our RBD imaging cohort 27.5% of patients have pathological nigrosome imaging, defined as absence of the DNH, compared with 7.7% of controls and 96% of patients with Parkinson's disease. Intriguingly, RBD patients with DNH absent did not have lower UPDRS-III scores than those with DNH present, but did have reduced dopamine transporter binding in the striatum, suggesting that nigral SWI may be able to identify individuals with dopaminergic decline[52]. This MRI method may have the potential to enrich cohorts for future neuroprotective trials in RBD, where participants with a high likelihood of conversion to motor Parkinson's are sought to achieve clinical endpoints in a manageable timeframe. However, longitudinal follow up to determine the true predictive value of SWI will be needed to confirm this.

Long-term follow-up

These baseline data already represent a rich source of data from a deeply phenotyped cohort. A key aspect of the OPDC-MRI cohort, however, is the longitudinal follow-up, which is ongoing. Clinical longitudinal data are acquired in the Discovery cohort every 18 months, and therefore, we will be able to use them to relate baseline imaging with clinical progression. This will potentially allow us to stratify patients and at-risk individuals and predict their progression. Information about conversion to Parkinson's of at-risk individuals will also be available, providing the ultimate validation of potential biomarkers.

At the latest clinical follow-up visit 2 PD participants have been re-diagnosed with multiple system atrophy (MSA), 3 with progressive supranuclear palsy (PSP) and 1 with motor neurone disease. For the remaining PD patients, the diagnosis was confirmed with diagnostic confidence over 90%, except one with 80% and one with 55%. Among RBD participants, 11 converted after receiving baseline MRI: 6 converted to PD, 2 to dementia with Lewy bodies, 2 to MSA and 1 to pure autonomic failure.

In 2015 we also commenced the acquisition of longitudinal follow-up MRI after 5 years from baseline for Parkinson's and HC, and after 2.5-3 years for RBD. This

For peer review only - http://bmjopen.bmj.com/site/about/guidelines.xhtml

difference in time after baseline was chosen because we predict that a number of the RBD patients will convert to Parkinson's, so we aim to collect multiple data points prior to conversion. In this way we hope to assess their trajectory during the prodromal phase and capture the associated brain changes. So far (December 2019) we have collected 91 follow-up scans: 47 iPD, 2 PD-LRRK2, 19 RBD, 2 RBD-GBA, 21 HC.

Strengths and limitations of this study

The main strength of this cohort is the collection of high quality 3T MRI data in a very well-phenotyped longitudinal cohort of Parkinson's and at-risk individuals. The RBD dataset is one of the biggest brain MRI datasets available for this population of at-risk individuals.

Inevitably, a limitation is due to the trade-off between depth of the phenotyping and size of the cohort. OPDC-MRI phenotyping is deep and longitudinal, however the size of the cohort is relatively modest, particularly when compared with population-level cohort studies.

We are also aware of the inevitable selection bias for this cohort. We recruited people already participating in OPDC Discovery cohort and therefore our cohort reflects the demographic of the catchment area. Moreover, our participants are those who were already participating in a research study and declared they were happy to be contacted for imaging (self-selection). The exclusion of participants who have severe motor dysfunction, more than mild head tremor or presence of dyskinesia/dystonia, means that the study includes only patients that were able to travel and tolerate an MRI scan "off" medication. As a result, our cohort cannot fully capture the variability of Parkinson's and RBD patients (e.g. patients with severe motor dysfunction may be less able to travel and tolerate an MRI scan; patients with comorbidities such as lung disease may find it hard to lie flat for the scan; severely obese patients cannot be scanned in our system; very apathetic patients may not respond to postal invites).

Another key strength of the OPDC-MRI cohort is the imaging protocol. All MRI data were acquired using the same MRI scanner, which is quite unique for a study of this duration. In this way all baseline data, as well as longitudinal MRI data will be truly comparable, with no effect due to scanner and/or protocol change. The protocol

For peer review only - http://bmjopen.bmj.com/site/about/guidelines.xhtml

includes both standard sequences, which are also comparable with other studies, as well as more experimental sequences acquired to investigate study-specific research questions.

We incorporated a trade-off between exploiting the latest techniques available in MRI and continuity of protocol throughout the study. While we could not use the most up-to-date sequences (e.g. our EPI images - dMRI and rfMRI - are not acquired with multiband acceleration), we managed to reach a balance by fixing the core sequences to maintain continuity, while changing the experimental sequences as the field evolved to include promising techniques as they became available.

As detailed in the previous section, the cohort will be enriched by collecting longitudinal follow-up MRI data. The challenge will be to be able to get data from those patients who have progressed quickly and will be in a more severe Parkinson's stage. While they may still be able to undergo a telephone interview or clinical assessment, they may not be willing or able to tolerate an MRI scan.

As described in more details in the "Collaboration and data sharing" section below, we have made statistical maps of our results publicly available, as well as support data relative to the analyses. The data presented here are also available to request (details below).

Collaboration and data sharing

Details about collaborating with OPDC can be found at https://www.opdc.ox.ac.uk/external-collaborations; OPDC is part of the CENTRE-PD twinning project (https://www.centre-pd.lu), we have ongoing international collaborations and are open for new proposals.

The data presented in this work (baseline imaging, demographics and clinical variables) will be available through the Dementias Platform UK (https://portal.dementiasplatform.uk), where data can be accessed by submitting a study proposal. Please note that longitudinal data will become available at a later stage.

Statistical maps are available on NeuroVault[59] for the following publications:

[11] https://neurovault.org/collections/2694/;

[30] https://identifiers.org/neurovault.collection:5448;

For peer review only - http://bmjopen.bmj.com/site/about/guidelines.xhtml

[31] (Results relative to a replication of the original study on an increased sample – see also supplementary figure S2): https://identifiers.org/neurovault.collection:5686;

[38] https://identifiers.org/neurovault.collection:2953

[57] https://identifiers.org/neurovault.collection:2681.

Other types of support data related to the analyses are available online (https://ora.ox.ac.uk/objects/uuid:8200af66-f438-4a7b-ad14-e8b032f0a9e7)[22] and the repository will keep being populated as the analyses progress.

For peer review only

Tables

Table 1. Demographic and clinical characteristics of the OPDC-MRI cohort.

	iPD	PD-LRRK2	PD-GBA	RBD	RBD-GBA	aGBA	HC
N	119	5	10	74	3	8	68
Age [years] (mean±stdev)	64.1 ±10.1	66.0 ±11.6	63.8 ±10.3	65.8 ±7.6	61.2 ±7.4	65.9 ±7.6	65.9 ±8.7
Gender [M/F]	76/43	2/3	6/4	68/6	3/0	3/5	45/23
Parkinson's disease duration at time of MRI [years] (mean±stdev)	(n=117) 2.31 ±1.52	3.06 ±3.49	3.29 ±1.81	-	-	-	-
Time between MRI and closest clinical assessment [days] (mean±stdev)	(n=118) 108 ±104	79 ± 105	140 ±107	115 ± 90	6 ± 4	(n=6) 273 ±25	(n=64) 387±644
Levodopa equivalent dose (LEDD) (mean±stdev) §	(n=117) 335±243	340 ±198	463 ±243	-	-	-	-
Hoen & Yahr (mean±stdev) §	(n=118) 1.79±0.57	2.40 ±0.89	2.10 ±0.88	(n=71) 0.03 ±0.17	0	(n=6) 0	(n=64) 0
UPDRS III (mean±stdev) § #	(n=118) 24.0 ±10.4	41.2 ±17.8	28.4 ±14.9	(n=74) 4.5 ±4.0	3.0 ±1.7	(n=6) 3.3 ±4.2	(n=64) 1.8 ±2.5
MoCA (mean±stdev) § ##	(n=117) 26.4 ±2.7	26.0 ±2.2	24.4 ±3.1	(n=73) 25.6 ±2.7	(n=2) 26.0 ±0.0	(n=6) 26.7 ±1.9	(n=62) 27.5 ±2.0

When data is missing for some participants, the number of values available is specified in brackets.

§ = Evaluated at closest clinical assessment (controls only have a baseline clinic visit at enrolment).

# = Corrected for missing questions using the approach described in [60].

= Corrected for education.

Legend: iPD = idiopathic Parkinson's patients; PD-LRRK2 = Parkinson's patients with mutation of the LRRK2 gene; PD-GBA = Parkinson's patients with mutation of the GBA gene; RBD = patients with REM sleep behavior disorder (RBD); RBD-GBA = RBD patients with pathogenic mutation of the GBA gene; aLRRK2 = asymptomatic carriers of a pathogenic mutation of the LRRK2 gene; aGBA = asymptomatic carriers of a pathogenic mutation of the GBA gene; HC = healthy controls.

For peer review only - http://bmjopen.bmj.com/site/about/guidelines.xhtml

Table 2. MRI core-sequences: parameters used in the study and number of available datasets for each modality.

	**T1**	**T1 WM nulled**	**T2-FLAIR**	**dMRI**	**rfMRI**
Sequence type	3D, MPRAGE	3D, MPRAGE	2D, FLAIR	EPI	EPI
Period of acquisition	2010-2018 (ongoing)				
TR (ms)	2040	3000	9000	9300	2000
TE (ms)	4.7	3.4	90	94	28
TI (ms)	900	409	2500	--	--
Flip angle (degrees)	8	8	150	--	89
Voxel size (mm^3)	1x1x1	0.9x0.9x1	1.1x0.9x3	2x2x2	3x3x3.5
FoV read (mm)	192	240	220	192	192
Fov phase (%)	90.6	81.3	100	100	100
Base Resolution	192	256	256	96	64
Phase Resolution (%)	100	100	75	100	100
Bandwidth (Hz/Px)	130	130	201	1628	2368
Orientation	Transversal	Transversal	Transversal	Transversal	Transversal
N volumes	--	--	--	60 directions + 5 b=0	180
Other sequence-specific characteristics	--	--	--	b-value = 1000 s/mm^2; Echo spacing = 0.69 ms	Echo spacing = 0.49 ms; Eyes open
Acquisition time	5 m 56 s	9 m 8 s	5 m 8 s	11 m 11 s	6 m 4 s
**Number of subjects**					
N iPD	119	118	100	114	117
N PD-LRRK2	5	5	1	2	4
N PD-GBA	10	10	1	10	10
N RBD	74	74	68	74	74
N RBD-GBA	3	3	3	3	3
N aGBA	8	8	6	8	8
N HC	68	66	55	66	68
N total	287	284	234	277	284

Legend: iPD = idiopathic Parkinson's patients; PD-LRRK2 = Parkinson's patients with mutation of the LRRK2 gene; PD-GBA = Parkinson's patients with mutation of the GBA gene; RBD = patients with REM sleep behavior disorder (RBD); RBD-GBA = RBD patients with pathogenic mutation of the GBA gene; aLRRK2 = asymptomatic carriers of a pathogenic mutation of the LRRK2 gene; aGBA = asymptomatic carriers of a pathogenic mutation of the GBA gene; HC = healthy controls.

For peer review only - http://bmjopen.bmj.com/site/about/guidelines.xhtml

Table 3. MRI experimental sequences: parameters used in the study and number of available datasets for each modality.

	**Neuromelanin-sensitive (NM-MRI)**	**SWI**
Sequence type	2D, T1 with MTR[#]	3D, T2*-weighted
Period of acquisition	From 2016, ongoing	
TR (ms)	1400	27
TE (ms)	17	20
Flip angle (degrees)	180	15
Voxel size (mm)	0.8 x 0.8 x 2	0.9 x 0.9 x1.5
FoV read (mm)	200	220
Fov phase (%)	100	90.6
Base Resolution	256	256
Phase Resolution (%)	100	96
Bandwidth (Hz/Px)	257	120
Orientation	Transversal	Transversal
Other sequence-specific characteristics	reduced FOV[$]; GRAPPA (accel factor 2)	Whole brain, GRAPPA (accel factor 2)
Acquisition time	3 m 41 s	4 m 54 s
**Number of subjects**		
N iPD	28	30
N PD-LRRK2	1	1
N PD-GBA	0	0
N RBD	45	46
N RBD-GBA	1	1
N aGBA	0	0
N HC	31	37
N total	106	115

[#] sequence adapted from Schwarz et al., 2011 [43]; [$] 24 slices covering the substantia nigra and locus coeruleus. When needed, the number of slices was reduced to remain within the specific absorption rate (SAR) limits without altering other acquisition parameters. Legend: iPD = idiopathic Parkinson's patients; PD-LRRK2 = Parkinson's patients with mutation of the LRRK2 gene; PD-GBA = Parkinson's patients with mutation of the GBA gene; RBD = patients with REM sleep behavior disorder (RBD); RBD-GBA = RBD patients with pathogenic mutation of the GBA gene; aLRRK2 = asymptomatic carriers of a pathogenic mutation of the LRRK2 gene; aGBA = asymptomatic carriers of a pathogenic mutation of the GBA gene; HC = healthy controls.

For peer review only - http://bmjopen.bmj.com/site/about/guidelines.xhtml

Figure legends

Figure 1. Structural sequences and related processing. T1weighted MRI (a) is brain extracted and bias field corrected (b) to perform tissue-type segmentation (GM in red, WM in yellow, CSF in blue) (c) and subcortical structures segmentation (d). T1-weighted white matter nulled (e) allows better contrast in the subcortical structures, which allows, for example, the segmentation of the Globus Pallidus (zoom) into its internal (GPi, yellow) and external (GPe, light blue) portions (f). FLAIR images (g) are used to detect and quantify white matter hyperintensities (red) (h).

Figure 2. Novel sequences: Neuromelanin-sensitive (NM-MRI, left) and susceptibility weighted imaging (SWI, right). Examples of segmentation of (a) the substantia nigra and (b) the locus coeruleus as hyperintense areas on NM-MRI. (c) Examples of the presence (top) absence (bottom) of the 'swallow tail' sign on SWI.

Funding Statement

The work was supported by the Monument Trust Discovery Award from Parkinson's UK (J-1403) and by the Wellcome Centre for Integrative Neuroimaging, the MRC Dementias Platform UK (MR/L023784/2), the National Institute for Health Research (NIHR) Oxford Biomedical Research Centre (BRC), and the NIHR Oxford Health BRC (a partnership between Oxford Health NHS Foundation Trust and the University of Oxford). The views expressed are those of the authors and not necessarily those of the NHS, the NIHR or the Department of Health.

JCK acknowledges support from the NIHR Oxford Health Clinical Research Facility. MR received funding support from a NIHR Academic Clinical Lectureship and a NIHR Oxford BRC Doctoral Training Fellowship. TRB received funding support from a Wellcome Trust Doctoral Training Fellowship, and a Biomedical Research Council Career Development Fellowship.

Acknowledgments

The authors would like to acknowledge all the participants and their families for participating in the study. They also thank Tim Quinnell, Oliver Bandmann, Gary Dennis, Zenobia Zaiwalla and Graham Lennox for patients recruitment and in-clinic

For peer review only

data collection. They are thankful to the staff of the Oxford Centre for Magnetic Resonance (OCMR), in particular Jane Francis, Kathryn Lacey, Rebecca Mills and Steven Knight, and to Amandine Louvel and Katie Ahmed for administering the cohort.

Footnotes

Author Contributions

LG had a major role in data acquisition and analysis, interpreted the data and drafted the manuscript for intellectual content.

JCK had a major role in data acquisition and analysis, interpreted the data and contributed to major revisions of the manuscript for intellectual content.

KSK, RALM, MR and TRB had a major role in data acquisition and analysis, interpreted the data and revised the manuscript for intellectual content.

ML, SE, FB, had a major role in data analysis and revised the manuscript for intellectual content.

MC and JR had a major role in data acquisition and revised the manuscript for intellectual content.

RWM, MTU, and CEM designed and conceptualised the study, interpreted the data and revised the manuscript for intellectual content.

All authors reviewed, critically revised and approved the manuscript.

Conflicts of interest

MTH reports grants from Parkinson's UK Monument Discovery Award during the conduct of the study; other from Biogen Digital and Roche Prodromal Advisory Boards, outside the submitted work. Other authors have nothing to declare.

Data Sharing Statement

The data presented in this work will be available through the Dementias Platform UK (https://portal.dementiasplatform.uk), where data can be accessed by submitting a study proposal. Statistical maps are available on NeuroVault.org and support data related to the analyses are available online (https://ora.ox.ac.uk/objects/uuid:8200af66-f438-4a7b-ad14-e8b032f0a9e7).

For peer review only

References

1. Oxford Parkinson's Disease Centre (OPDC) [Available from: https://opdc.medsci.ox.ac.uk/home.
2. Szewczyk-Krolikowski K, Tomlinson P, Nithi K, et al. The influence of age and gender on motor and non-motor features of early Parkinson's disease: initial findings from the Oxford Parkinson Disease Center (OPDC) discovery cohort. *Parkinsonism Relat Disord* 2014;20(1):99-105. doi: 10.1016/j.parkreldis.2013.09.025
3. Lawton M, Baig F, Rolinski M, et al. Parkinson's Disease Subtypes in the Oxford Parkinson Disease Centre (OPDC) Discovery Cohort. *J Parkinsons Dis* 2015;5(2):269-79. doi: 10.3233/JPD-140523
4. Barber TR, Lawton M, Rolinski M, et al. Prodromal Parkinsonism and Neurodegenerative Risk Stratification in REM Sleep Behavior Disorder. *Sleep* 2017;40(8) doi: 10.1093/sleep/zsx071
5. Vaillancourt DE, Spraker MB, Prodoehl J, et al. High-resolution diffusion tensor imaging in the substantia nigra of de novo Parkinson disease. *Neurology* 2009;72(16):1378-84. doi: 10.1212/01.wnl.0000340982.01727.6e
6. Pyatigorskaya N, Gallea C, Garcia-Lorenzo D, et al. A review of the use of magnetic resonance imaging in Parkinson's disease. *Ther Adv Neurol Disord* 2014;7(4):206-20. doi: 10.1177/1756285613511507
7. Tuite P. Brain Magnetic Resonance Imaging (MRI) as a Potential Biomarker for Parkinson's Disease (PD). *Brain Sci* 2017;7(6) doi: 10.3390/brainsci7060068
8. Helmich RC, Vaillancourt DE, Brooks DJ. The Future of Brain Imaging in Parkinson's Disease. *J Parkinsons Dis* 2018;8(s1):S47-S51. doi: 10.3233/JPD-181482
9. Barber TR, Klein JC, Mackay CE, et al. Neuroimaging in pre-motor Parkinson's disease. *Neuroimage Clin* 2017;15:215-27. doi: 10.1016/j.nicl.2017.04.011
10. Hughes AJ, Daniel SE, Kilford L, et al. Accuracy of clinical diagnosis of idiopathic Parkinson's disease: a clinico-pathological study of 100 cases. *Journal of neurology, neurosurgery, and psychiatry* 1992;55(3):181-4.
11. Szewczyk-Krolikowski K, Menke RA, Rolinski M, et al. Functional connectivity in the basal ganglia network differentiates PD patients from controls. *Neurology* 2014;83(3):208-14. doi: 10.1212/wnl.0000000000000592 [published Online First: 2014/06/13]
12. Sateia MJ. International Classification of Sleep Disorders-Third Edition Highlights and Modifications. *Chest* 2014;146(5):1387-94. doi: 10.1378/chest.14-0970
13. Rolinski M, Szewczyk-Krolikowski K, Tomlinson PR, et al. REM sleep behaviour disorder is associated with worse quality of life and other non-motor features in early Parkinson's disease. *Journal of neurology, neurosurgery, and psychiatry* 2014;85(5):560-6. doi: 10.1136/jnnp-2013-306104
14. Hanganu A, Monchi O. Structural Neuroimaging Markers of Cognitive Decline in Parkinson's Disease. *Parkinsons Dis* 2016;2016:3217960. doi: 10.1155/2016/3217960
15. Burton EJ, McKeith IG, Burn DJ, et al. Cerebral atrophy in Parkinson's disease with and without dementia: a comparison with Alzheimer's disease, dementia with Lewy bodies and controls. *Brain : a journal of neurology* 2004;127(Pt 4):791-800. doi: 10.1093/brain/awh088
16. Mak E, Su L, Williams GB, et al. Baseline and longitudinal grey matter changes in newly diagnosed Parkinson's disease: ICICLE-PD study. *Brain : a journal of neurology* 2015;138(Pt 10):2974-86. doi: 10.1093/brain/awv211
17. Zhang Y, Brady M, Smith S. Segmentation of brain MR images through a hidden Markov random field model and the expectation-maximization algorithm. *IEEE transactions on medical imaging* 2001;20(1):45-57. doi: 10.1109/42.906424 [published Online First: 2001/04/11]

For peer review only - http://bmjopen.bmj.com/site/about/guidelines.xhtml

18. Good CD, Johnsrude IS, Ashburner J, et al. A voxel-based morphometric study of ageing in 465 normal adult human brains. *NeuroImage* 2001;14(1 Pt 1):21-36. doi: 10.1006/nimg.2001.0786 [published Online First: 2001/08/30]
19. Patenaude B, Smith SM, Kennedy DN, et al. A Bayesian model of shape and appearance for subcortical brain segmentation. *NeuroImage* 2011;56(3):907-22. doi: 10.1016/j.neuroimage.2011.02.046
20. Visser E, Keuken MC, Douaud G, et al. Automatic segmentation of the striatum and globus pallidus using MIST: Multimodal Image Segmentation Tool. *NeuroImage* 2016;125:479-97. doi: 10.1016/j.neuroimage.2015.10.013
21. Griffanti L, Zamboni G, Khan A, et al. BIANCA (Brain Intensity AbNormality Classification Algorithm): A new tool for automated segmentation of white matter hyperintensities. *NeuroImage* 2016;141:191-205. doi: 10.1016/j.neuroimage.2016.07.018
22. OPDC MRI analysis [Available from: https://ora.ox.ac.uk/objects/uuid:8200af66-f438-4a7b-ad14-e8b032f0a9e7.
23. Andersson JLR, Sotiropoulos SN. An integrated approach to correction for off-resonance effects and subject movement in diffusion MR imaging. *NeuroImage* 2016;125:1063-78. doi: 10.1016/j.neuroimage.2015.10.019
24. Andersson JLR, Graham MS, Zsoldos E, et al. Incorporating outlier detection and replacement into a non-parametric framework for movement and distortion correction of diffusion MR images. *NeuroImage* 2016;141:556-72. doi: 10.1016/j.neuroimage.2016.06.058
25. Andersson JLR, Graham MS, Drobnjak I, et al. Towards a comprehensive framework for movement and distortion correction of diffusion MR images: Within volume movement. *NeuroImage* 2017;152:450-66. doi: 10.1016/j.neuroimage.2017.02.085
26. Basser PJ, Mattiello J, LeBihan D. Estimation of the effective self-diffusion tensor from the NMR spin echo. *J Magn Reson B* 1994;103(3):247-54.
27. Pierpaoli C, Jezzard P, Basser PJ, et al. Diffusion tensor MR imaging of the human brain. *Radiology* 1996;201(3):637-48. doi: 10.1148/radiology.201.3.8939209
28. Smith SM, Fox PT, Miller KL, et al. Correspondence of the brain's functional architecture during activation and rest. *Proceedings of the National Academy of Sciences of the United States of America* 2009;106(31):13040-5. doi: 10.1073/pnas.0905267106 [published Online First: 2009/07/22]
29. Robinson S, Basso G, Soldati N, et al. A resting state network in the motor control circuit of the basal ganglia. *BMC neuroscience* 2009;10:137. doi: 10.1186/1471-2202-10-137 [published Online First: 2009/11/26]
30. Rolinski M, Griffanti L, Szewczyk-Krolikowski K, et al. Aberrant functional connectivity within the basal ganglia of patients with Parkinson's disease. *Neuroimage Clin* 2015;8:126-32. doi: 10.1016/j.nicl.2015.04.003
31. Rolinski M, Griffanti L, Piccini P, et al. Basal ganglia dysfunction in idiopathic REM sleep behaviour disorder parallels that in early Parkinson's disease. *Brain : a journal of neurology* 2016;139(Pt 8):2224-34. doi: 10.1093/brain/aww124
32. Beckmann CF, Smith SM. Probabilistic independent component analysis for functional magnetic resonance imaging. *IEEE transactions on medical imaging* 2004;23(2):137-52. doi: 10.1109/tmi.2003.822821 [published Online First: 2004/02/18]
33. Griffanti L, Salimi-Khorshidi G, Beckmann CF, et al. ICA-based artefact removal and accelerated fMRI acquisition for improved resting state network imaging. *NeuroImage* 2014;95:232-47. doi: 10.1016/j.neuroimage.2014.03.034 [published Online First: 2014/03/25]
34. Salimi-Khorshidi G, Douaud G, Beckmann CF, et al. Automatic denoising of functional MRI data: combining independent component analysis and hierarchical fusion of classifiers. *NeuroImage* 2014;90:449-68. doi: 10.1016/j.neuroimage.2013.11.046 [published Online First: 2014/01/07]
35. Jenkinson M, Smith S. A global optimisation method for robust affine registration of brain images. *Med Image Anal* 2001;5(2):143-56.

For peer review only

36. Andersson JL, Jenkinson M, Smith S. Non-linear registration aka Spatial normalisation FMRIB Technial Report TR07JA2. *FMRIB Analysis Group of the University of Oxford* 2007
37. Filippini N, MacIntosh BJ, Hough MG, et al. Distinct patterns of brain activity in young carriers of the APOE-epsilon4 allele. *Proceedings of the National Academy of Sciences of the United States of America* 2009;106(17):7209-14. doi: 10.1073/pnas.0811879106 [published Online First: 2009/04/10]
38. Griffanti L, Rolinski M, Szewczyk-Krolikowski K, et al. Challenges in the reproducibility of clinical studies with resting state fMRI: An example in early Parkinson's disease. *NeuroImage* 2016;124(Pt A):704-13. doi: 10.1016/j.neuroimage.2015.09.021
39. Sasaki M, Shibata E, Tohyama K, et al. Neuromelanin magnetic resonance imaging of locus ceruleus and substantia nigra in Parkinson's disease. *Neuroreport* 2006;17(11):1215-8. doi: 10.1097/01.wnr.0000227984.84927.a7
40. Chen X, Huddleston DE, Langley J, et al. Simultaneous imaging of locus coeruleus and substantia nigra with a quantitative neuromelanin MRI approach. *Magnetic resonance imaging* 2014;32(10):1301-6. doi: 10.1016/j.mri.2014.07.003
41. Braak H, Del Tredici K. Invited Article: Nervous system pathology in sporadic Parkinson disease. *Neurology* 2008;70(20):1916-25. doi: 10.1212/01.wnl.0000312279.49272.9f
42. Zucca FA, Segura-Aguilar J, Ferrari E, et al. Interactions of iron, dopamine and neuromelanin pathways in brain aging and Parkinson's disease. *Prog Neurobiol* 2017;155:96-119. doi: 10.1016/j.pneurobio.2015.09.012
43. Schwarz ST, Rittman T, Gontu V, et al. T1-weighted MRI shows stage-dependent substantia nigra signal loss in Parkinson's disease. *Mov Disord* 2011;26(9):1633-8. doi: 10.1002/mds.23722
44. Sulzer D, Cassidy C, Horga G, et al. Neuromelanin detection by magnetic resonance imaging (MRI) and its promise as a biomarker for Parkinson's disease. *NPJ Parkinsons Dis* 2018;4:11. doi: 10.1038/s41531-018-0047-3
45. Ehrminger M, Latimier A, Pyatigorskaya N, et al. The coeruleus/subcoeruleus complex in idiopathic rapid eye movement sleep behaviour disorder. *Brain : a journal of neurology* 2016;139(Pt 4):1180-8. doi: 10.1093/brain/aww006
46. Knudsen K, Fedorova TD, Hansen AK, et al. In-vivo staging of pathology in REM sleep behaviour disorder: a multimodality imaging case-control study. *The Lancet Neurology* 2018;17(7):618-28. doi: 10.1016/S1474-4422(18)30162-5
47. Pyatigorskaya N, Gaurav R, Arnaldi D, et al. Magnetic Resonance Imaging Biomarkers to Assess Substantia Nigra Damage in Idiopathic Rapid Eye Movement Sleep Behavior Disorder. *Sleep* 2017;40(11) doi: 10.1093/sleep/zsx149
48. Neuromelanin-MRI in REM sleep behaviour disorder predicts
dopaminergic dysfunction and clinical risk of Parkinson's disease - abstract n.176. International Parkinson and Movement Disorder Society; 2018. Movement Disorders.
49. Haacke EM, Mittal S, Wu Z, et al. Susceptibility-weighted imaging: technical aspects and clinical applications, part 1. *AJNR American journal of neuroradiology* 2009;30(1):19-30. doi: 10.3174/ajnr.A1400
50. Mahlknecht P, Krismer F, Poewe W, et al. Meta-analysis of dorsolateral nigral hyperintensity on magnetic resonance imaging as a marker for Parkinson's disease. *Mov Disord* 2017;32(4):619-23. doi: 10.1002/mds.26932
51. Schwarz ST, Afzal M, Morgan PS, et al. The 'swallow tail' appearance of the healthy nigrosome - a new accurate test of Parkinson's disease: a case-control and retrospective cross-sectional MRI study at 3T. *PloS one* 2014;9(4):e93814. doi: 10.1371/journal.pone.0093814
52. Barber TR, Griffanti L, Bradley KM, et al. Nigrosome 1 imaging in REM sleep behavior disorder and its association with dopaminergic decline. *Ann Clin Transl Neurol* 2020;7(1):26-35. doi: 10.1002/acn3.50962

For peer review only

53. Menke RA, Szewczyk-Krolikowski K, Jbabdi S, et al. Comprehensive morphometry of subcortical grey matter structures in early-stage Parkinson's disease. *Human brain mapping* 2014;35(4):1681-90. doi: 10.1002/hbm.22282
54. Klein JC, Rolinski M, Griffanti L, et al. Cortical structural involvement and cognitive dysfunction in early Parkinson's disease. *NMR in biomedicine* 2018;31(4):e3900. doi: 10.1002/nbm.3900
55. Minett T, Su L, Mak E, et al. Longitudinal diffusion tensor imaging changes in early Parkinson's disease: ICICLE-PD study. *J Neurol* 2018;265(7):1528-39. doi: 10.1007/s00415-018-8873-0
56. Zamboni G, Wilcock GK, Douaud G, et al. Resting functional connectivity reveals residual functional activity in Alzheimer's disease. *Biological psychiatry* 2013;74(5):375-83. doi: 10.1016/j.biopsych.2013.04.015 [published Online First: 2013/06/04]
57. Griffanti L, Stratmann P, Rolinski M, et al. Exploring variability in basal ganglia connectivity with functional MRI in healthy aging. *Brain Imaging Behav* 2018;12(6):1822-27. doi: 10.1007/s11682-018-9824-1
58. Arya Z, Griffanti L, Mackay CE, et al. Iterative Dual LDA: A Novel Classification Algorithm for Resting State fMRI. *Lect Notes Comput Sc* 2016;10019:279-86. doi: 10.1007/978-3-319-47157-0_34
59. Gorgolewski KJ, Varoquaux G, Rivera G, et al. NeuroVault.org: a web-based repository for collecting and sharing unthresholded statistical maps of the human brain. *Front Neuroinform* 2015;9:8. doi: 10.3389/fninf.2015.00008
60. Goetz CG, Luo S, Wang L, et al. Handling missing values in the MDS-UPDRS. *Mov Disord* 2015;30(12):1632-8. doi: 10.1002/mds.26153

For peer review only

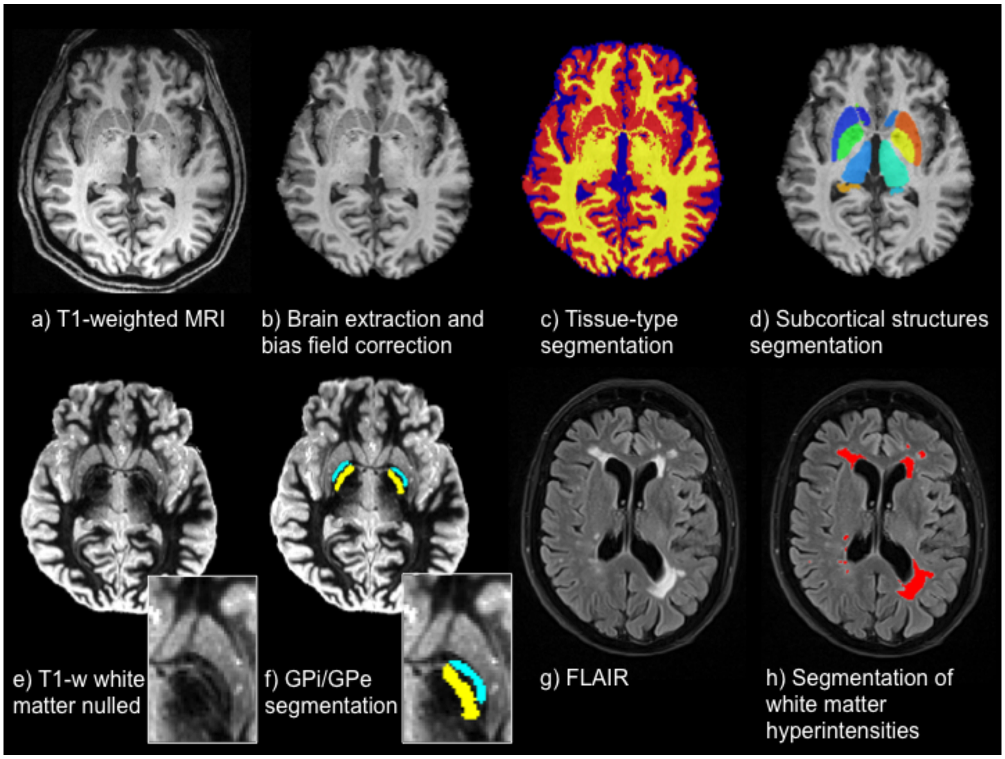

Structural sequences and related processing. T1weighted MRI (a) is brain extracted and bias field corrected (b) to perform tissue-type segmentation (GM in red, WM in yellow, CSF in blue) (c) and subcortical structures segmentation (d). T1-weighted white matter nulled (e) allows better contrast in the subcortical structures, which allows, for example, the segmentation of the Globus Pallidus (zoom) into its internal (GPi, yellow) and external (GPe, light blue) portions (f). FLAIR images (g) are used to detect and quantify white matter hyperintensities (red) (h).

254x190mm (72 x 72 DPI)

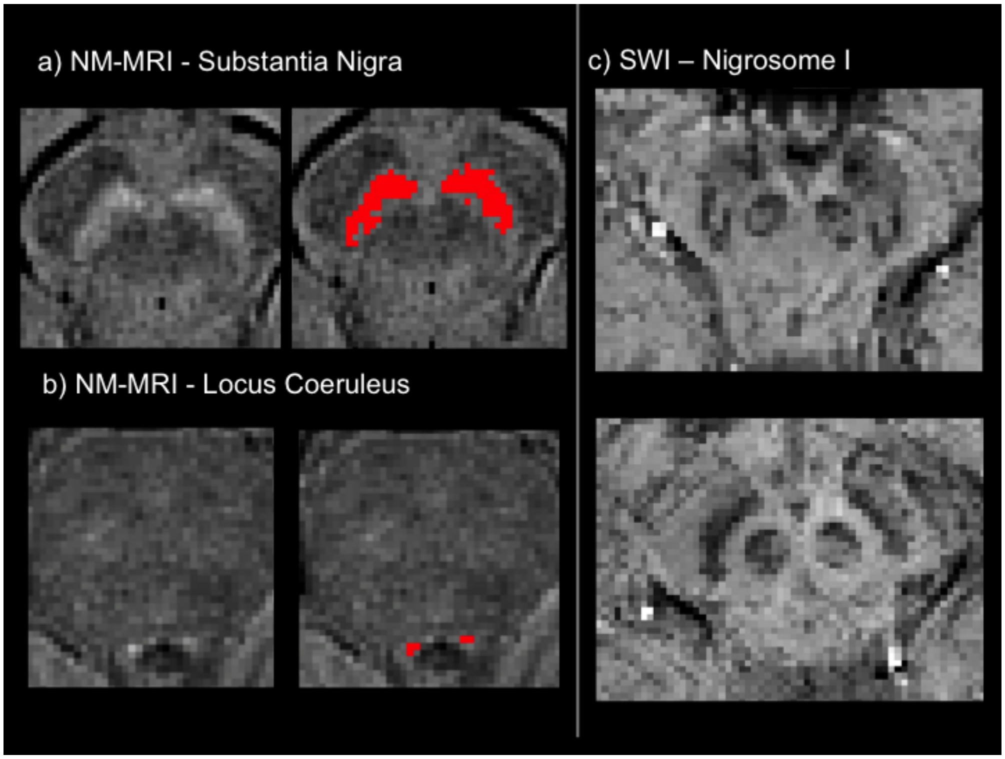

Novel sequences: Neuromelanin-sensitive (NM-MRI, left) and susceptibility weighted imaging (SWI, right). Examples of segmentation of (a) the substantia nigra and (b) the locus coeruleus as hyperintense areas on NM-MRI. (c) Examples of the presence (top) absence (bottom) of the 'swallow tail' sign on SWI.

254x190mm (72 x 72 DPI)

<u>Supplementary material</u> - Cohort Profile: the Oxford Parkinson's Disease Centre Discovery Cohort Magnetic Resonance Imaging sub-study (OPDC-MRI).

Griffanti et al.

Supplementary Table S1

Questionnaires administered to the OPDC Discovery cohort participants and derived variables.

Patient-Completed Questionnaires	
**Demographics**	Basic demographic information
**Big Five Inventory**[1]	Personality profiling
**Epworth Sleepiness Scale**[2]	Daytime sleepiness questionnaire
Sleepiness, (%)	dichotomised to 'unusual level of sleepiness - ESS >=11' versus 'normal results - EPS <=10'
**RBD Questionnaire**[3]	Patient reported features of RBD
RBD, (%)	dichotomised to 'positive result - RBD >=5' versus 'normal - RBD<=4'
**Health-Related Quality of Life EQ5D and EQ-VAS**[4]	General assessment of quality of life
Pain, EQ5D, (%)	pain score from EQ-5D health questionnaire dichotomised to 'no pain or discomfort' versus 'moderate or extreme pain or discomfort'
**Constipation Questionnaire**	Assessment of frequency of bowel movements
Constipation, (%)	average daily bowel movement frequency in the past week dichotomised to 'constipation - <1' versus 'normal - >=1'
Use of laxatives, (%)	use of laxatives in the past week dichotomised to 'any' versus 'none'
**UPDRS I, Non-motor aspects of daily living**[5]	Assessment of basic non-motor symptoms in PD in the last week. Each item was dichotomised as ‘present – score 1-4’ versus ‘absent – score 0’.
**UPDRS II, Motor aspects of daily living**[5]	Assessment of basic motor symptoms in PD in the last week. Each item was dichotomised as ‘present – score 1-4’ versus ‘absent – score 0’.
**Social background**	Information about education, wealth and employment.
Accommodation at diagnosis, (own, %)	Dichotomised to ‘own’ versus ‘renting’
Bedrooms at diagnosis, (≤3, %)	Dichotomised to ‘≤3’ versus ‘>3’
Vehicles at diagnosis, (≤1, %)	Dichotomised to ‘≤1’ versus >1’
Employment at diagnosis, (employed, %)	Dichotomised to 'employed in any capacity' versus not employed
Last job, (managing people, %)	Dichotomised to 'job involving managing people' versus 'job not involving managing people'
**Leeds Depression and Anxiety General Scales**[6]	Assessment of level of depression and anxiety in undiagnosed cases.

Depression, Leeds SAD General, (%)	dichotomised to 'case - >=7' versus 'non-case - <7'[6]
Anxiety, Leeds SAA General, (%)	dichotomised to 'case - >=7' versus 'non-case - <7'[6]
**Beck Depression Inventory (BDI)**[7]	Extensive assessment of symptoms of depression.
**Questionnaire for Impulsive-Compulsive Disorders in Parkinson's Disease (QUIP Anytime Short)**[8]	Assessment of impulsive and compulsive disorders in PD occurring anytime in life.
Any ICD, QUIP, (%)	dichotomised score for any main impulsive disorder (gambling, sex, buying or eating)
Any other OCD, QUIP, (%)	dichotomised score for any other compulsive behaviour (organised activities, ordering, purposeless walking or driving)
Overmedicating, QUIP, (%)	dichotomised score for any excessive parkinsonian medication use
Nurse-Administered Questionnaires	
**General Exam**	Weight, height, lying and standing blood pressure.
BMI	Body Mass Index calculated as: weight in kg divided by square of height in meters.
Orthostatic hypotension, (%)	Orthostatic hypotension defined as difference between lying and standing blood pressure greater than 20 (SBP) or 10 (DBP).
**MOCA**[9]	Assessment of general cognition. Additional point was added for patients with <=12 years of education.
Cognitive impairment, (normal:MCI:dementia, %)	Defined based on MOCA scores: normal cognition 26-30, MCI 21-25, dementia <=20[10-12]
**MMSE**[13]	Assessment of general cognition.
**Phonemic and Semantic Fluency**	Assessment of fluency scored according to population norms.
**Peg board tests**[14]	Test of manual dexterity. Test results indicate number of pegs/items inserted into peg board within 30s (1min for 'Arm coordination')
Arm asymmetry (absolute value of right score - left score), Peg board, (mean±SD)	absolute difference between right and left hand scores reflecting asymmetry of disease
**Get-up-and-go**[15]	Test of walking speed. The result is an average in seconds of three trials of timed 3m walk.
**Flamingo test** [16]	Test of balancing on one leg for 30s. The result is dichotomised to 'subjects who completed 30s trial' versus 'subjects who failed before 30s'.
Doctor Administered Questionnaires	
**Past Medical History**	Review of medical history covering most common conditions.
Vascular risk factors, (%)	Percentage of patients with any of the following: high blood pressure, high cholesterol level, diabetes mellitus
Cerebro-vascular disease, (%)	Percentage of patients with any of the following: stroke or TIA
Cardio-vascular disease, (%)	Percentage of patients with any of the following: angina, heart attack, heart failure

For peer review only - http://bmjopen.bmj.com/site/about/guidelines.xhtml

Cancers, (%)	Percentage of patients with any of the following: lung, bowel, breast, prostate, melanoma, or any other cancer reported by patient
Respiratory disease, (%)	Percentage of patients with any of the following: asthma, chronic bronchitis, emphysema
**Mini Environmental Risk Questionnaire for PD Baseline (MERQ-PD B)**[17]	Assessment for exposure to commonly accepted risk factors for PD.
Smoking before PD, (%)	Dichotomised to 'smoking at least one cigarette per day for at least 6 months' versus 'non-smoker by that definition'.
Alcohol consumption before PD, (%)	Dichotomised to 'drinking any alcohol' versus 'non-drinker'.
Medication (different types) before PD	Dichotomised to 'any use' versus 'no use'.
**Modified Schwab & England Activities of Daily Living**	Assessment of activities of daily living.
**PD Features**[17]	Presence of typical features of PD at diagnosis as reported by the patient and in the notes.
**Family History**[17]	Extensive family history including occurrence of PD and other neurological disorders in the family.
**PD Medication**	List of current and previous medication taken for PD.
Levodopa equivalent daily dose (LEDD)[18]	Calculated from all current dopaminergic medication.
**Clinical Global Impression of Change (CGI)**	Eight-level scale assessing change in symptoms after starting PD medication.
**Other Medication**[17]	List of medication unrelated to PD.
**Sniffin Sticks**[19]	Assessment of hyposmia. Sniffin Sticks was score dichotomised at 10th percentile according to normal values for age and sex.
**UPDRS I Non-motor features of daily living**[5]	Clinician-assessed non-motor symptoms of PD.
Fatigue in PD, (%)	Dichotomised score from item 1.13 of MDS-UPDRS I: 'any fatigue' versus 'no fatigue'
Apathy in PD, (%)	Dichotomised score from item 1.5 of MDS-UPDRS I: 'any apathy' versus 'no apathy'
Hallucinations in PD, (%)	Dichotomised score from item 1.2 of MDS-UPDRS I: 'any hallucinations' versus 'no hallucinations'
**UPDRS III Motor Examination**[5]	Standardised assessment of PD signs.
Rigidity score (score/number of items)	Sum of scores from item 3.3 MDS-UPDRS III divided by 5
Bradykinesia scores, (score/number of items)	sum of scores from items 3.4, 3.5, 3.6, 3.7, 3.8, divided by 10
Postural scores, (score/number of items)	Sum of scores from items 3.9, 3.10, 3.11, 3.12, 3.13, divided by 5

Tremor scores, (score/number of items)	Sum of scores from items 3.15, 3.16, 3.17, divided by 9
Motor subtype, (PIGD:Mix:Tremor, %)	Sum of scores from all MDS-UPDRS tremor items (2.10, 3.15, 3.16, 3.17) divided by the sum of the postural and gait items (2.12, 2.13, 3.10, 3.11, 3.12) gives a Tremor/PIGD index; index <=1 defines PIGD subtype, >=1.5 - tremor dominant subtype, between 1-1.5 - mixed subtype[20]
Face and neck score, (score/number of items)	Sum of scores from MDS-UPDRS III items for face and neck symptoms (3.1, 3.2, 3.3 Neck, 3.17 Lip/Jaw) divided by 4
Arms score, (score/number of items)	Sum of scores from MDS-UPDRS III items for upper extremities (3.3 RUE, 3.3 LUE, 3.4, 3.5, 3.6, 3.15, 3.16, 3.17 RUE, 3.17 LUE) divided by 14
Legs score, (score/number of items)	Sum of scores from MDS-UPDRS III items for lower extremities (3.3 RLE, 3.3 LLE, 3.7, 3.8, 3.17 RLE, 3.17 LLE) divided by 8
Symptom laterality, (right : left : symmetrical, %)	Right-dominant symptoms: difference between all right-sided scores and all left-sided scores >=4; left-dominant symptoms: difference between all left-sided scores and all right-sided scores >=4; symmetrical: intermediate scores[21]
Annualised UPDRS III (UPDRS III/disease duration)	Total UPDRS III score divided by disease duration in years since symptom onset, reflecting symptomatic progression since disease onset
**UPDRS IV Motor Complications**[5]	Assessment of dyskinesia, motor fluctuations and dystonia.
Presence of Dyskinesia, (%)	Dichotomised score from item 4.1 of MDS-UPDRS IV: 'No dyskinesia' versus 'Any dyskinesia'
Presence of Motor Fluctuations, (%)	Dichotomised score from item 4.3 of MDS-UPDRS IV: 'No OFF time' versus 'Any OFF time'
**Specific Clinical Features**[17]	Assessment of atypical parkinsonian features not fitting with idiopathic PD.
Sexual dysfunction, (%)	Significant change in sexual functioning in the past year
Erectile dysfunction, (%)	Ability to maintain erection without treatment dichotomised to 'very poor or poor' versus 'fair, good or very good'
**Likelihood of Diagnosis**	Clinician-assessed likelihood of diagnosis of idiopathic PD from 0%-100%.

References for Supplementary Table S1

1. John, O. P. & Srivastava, S. The Big Five trait taxonomy: History, measurement, and theoretical perspectives. *Handbook of personality: Theory and research* **2,** 102–138 (1999).
2. Johns, M. W. A new method for measuring daytime sleepiness: the Epworth sleepiness scale. *Sleep* **14,** 540–545 (1991).
3. Stiasny-Kolster, K. *et al.* The REM sleep behavior disorder screening questionnaire--a

new diagnostic instrument. *Mov Disord* **22,** 2386–2393 (2007).
4. EuroQol--a new facility for the measurement of health-related quality of life. The EuroQol Group. *Health Policy* **16,** 199–208 (1990).
5. Goetz, C. G. *et al.* Movement Disorder Society-sponsored revision of the Unified Parkinson's Disease Rating Scale (MDS-UPDRS): Process, format, and clinimetric testing plan. *Mov Disord* **22,** 41–47 (2007).
6. Snaith, R. P., Bridge, G. W. & Hamilton, M. The Leeds scales for the self-assessment of anxiety and depression. *Br J Psychiatry* **128,** 156–165 (1976).
7. Beck, A., Ward, C. & Mendelson, M. Beck depression inventory (BDI). *Archives of General Psychiatry* (1961).
8. Weintraub, D. *et al.* Validation of the questionnaire for impulsive-compulsive disorders in Parkinson's disease. *Mov Disord* **24,** 1461–1467 (2009).
9. Nasreddine, Z. S. *et al.* The Montreal Cognitive Assessment, MoCA: a brief screening tool for mild cognitive impairment. *J Am Geriatr Soc* **53,** 695–699 (2005).
10. Dalrymple-Alford, J. C. *et al.* The MoCA: Well-suited screen for cognitive impairment in Parkinson disease. *Neurology* **75,** 1717–1725 (2010).
11. Gill, D. J., Freshman, A., Blender, J. A. & Ravina, B. The Montreal cognitive assessment as a screening tool for cognitive impairment in Parkinson's disease. *Mov Disord* **23,** 1043–1046 (2008).
12. Nazem, S. *et al.* Montreal cognitive assessment performance in patients with Parkinson's disease with "normal" global cognition according to mini-mental state examination score. *J Am Geriatr Soc* **57,** 304–308 (2009).
13. Folstein, M. F., Folstein, S. E. & McHugh, P. R. 'Mini-mental state'. A practical method for grading the cognitive state of patients for the clinician. *J Psychiatr Res* **12,** 189–198 (1975).
14. Desrosiers, J., Hébert, R., Bravo, G. & Dutil, E. The Purdue Pegboard Test: normative data for people aged 60 and over. *Disabil Rehabil* **17,** 217–224 (1995).
15. Shumway-Cook, A., Brauer, S. & Woollacott, M. Predicting the probability for falls in community-dwelling older adults using the Timed Up & Go Test. *Phys Ther* **80,** 896–903 (2000).
16. Tsigilis, N., Douda, H. & Tokmakidis, S. P. Test-retest reliability of the Eurofit test battery administered to university students. *Percept Mot Skills* **95,** 1295–1300–1300 (2002).
17. http://www.commondataelements.ninds.nih.gov
18. Tomlinson, C. L. *et al.* Systematic review of levodopa dose equivalency reporting in Parkinson's disease. *Mov Disord* **25,** 2649–2653 (2010).
19. Hummel, T., Kobal, G., Gudziol, H. & Mackay-Sim, A. Normative data for the 'Sniffin' Sticks' including tests of odor identification, odor discrimination, and olfactory thresholds: an upgrade based on a group of more than 3,000 subjects. *Eur Arch Otorhinolaryngol* **264,** 237–243 (2007).
20. Skeie, G. O., Muller, B., Haugarvoll, K., Larsen, J. P. & Tysnes, O. B. Differential effect of environmental risk factors on postural instability gait difficulties and tremor dominant Parkinson's disease. *Mov Disord* **25,** 1847–1852 (2010).
21. Uitti, R. J., Baba, Y., Whaley, N. R., Wszolek, Z. K. & Putzke, J. D. Parkinson disease: handedness predicts asymmetry. *Neurology* **64,** 1925–1930 (2005).

For peer review only

Supplementary Information

Discontinued sequences

Supplementary Table S2 illustrates the main acquisition parameters and the number of available subjects per group for each of the sequences that were acquired between 2010 and 2016.

The diffusion-weighted sequence with reduced FOV centered on the substantia nigra (SN) was inspired by the study by Vaillancourt and colleagues [1], who found reduced FA in the SN of subjects with Parkinson's compared with controls, especially in the caudal compared with the rostral region of interest. We acquired a similar sequence in our first 50 participants, but did not find any difference between Parkinson's and controls (unpublished). We therefore decided to discontinue the sequence.

DESPOT1 and DESPOT2 (driven equilibrium single pulse observation of T1and T2, respectively) are a set of rapid combined T1 and T2 mapping approaches developed by Deoni and colleagues to be acquired in a clinically acceptable time [2]. They also demonstrated that by acquiring DESPOT2 in addition to DESPOT1 it is possible to retrospectively calculate 'synthetic' weighted images with arbitrary T1 or T2 weighting, since the underlying relaxation parameters are known. A study using quantitative MRI (qMRI) in early Parkinson's [3] found widespread reduction of midbrain T1 values contralateral to the clinically more severely affected limbs and suggested that T1 may be a valuable marker for the monitoring of progressive neuronal loss in Parkinson's. T1 qMRI probes the chemical composition of the tissues, providing complementary information to the microstructural one available with diffusion imaging. In light of these findings we experimented with this sequence in OPDC-MRI. Quantitative T1 maps were generated using DESPOT-HiFi (dual SPGR, 144 slices) with two IR-SPGR images to assess B1 field inhomogeneity[2]. While we were able to use the T1 maps obtained from DESPOT1[4], the quality of DESPOT2 obtained was not deemed sufficient to perform further analyses.

The study by Baudrexel and colleagues[3] also acquired multi-echo T2* and performed T2* mapping of the midbrain, which revealed a bilateral decrease of T2* values restricted to the SN, indicating a local increase in total iron content. They conclude that nigral T2* reductions might be associated with an increased general vulnerability for the development of the disorder. We therefore included this small FOV, multi ECHO T2* sequence to explore its diagnostic usefulness given that SN

For peer review only

iron accumulation seems implicated in Parkinson's and T2* correlates with iron content.

This sequence was subsequently replaced with whole brain SWI because, despite not being able to provide T2* mapping (non multi-echo), it has a much shorter acquisition time and covers the whole brain, allowing, for example, to quantify iron content in the basal ganglia (see main text for details).

Table S2. Discontinued MRI experimental sequences: parameters used in the study and number of available datasets for each modality.

	**SN dMRI**	**qT1**	**qT2**	**SN GRE**
Sequence type	EPI	3D, DESPOT-HiFi	3D, DESPOT-HiFi	2D, T2*-weighted
Period of acquisition	2010-2013	2010-2014	2010-2014	2010-2016
TR (ms)	3000	8	55	648
TE (ms)	82	3.7	2.8	5 to 60#
TI (ms)	--	--	--	--
Flip angle (degrees)	90	4, 18	15, 60	45
Voxel size (mm)	0.8x0.8x4	1.1x1.1x1.1	1.1x1.1x1.1	1 x 1 x 2
FoV read (mm)	60	270	270	256
Fov phase (%)	41.7	83.3	83.3	100
Base Resolution	72	240	240	256
Phase Resolution (%)	100	100	100	100
Bandwidth (Hz/Px)	938	210	605	260
Orientation	Transversal	Sagittal	Sagittal	Transversal
N volumes	30 directions + 1 b0 x 10 averages	--	--	--
Other sequence-specific characteristics	b-value = 600 s/mm; reduced FOV, 15 slices	dual SPGR, two IR-SPGR images§	RF Phase Increment = 0, 180	#12 TEs, 5 ms increment, reduced FOV, 10 slices
Acquisition time	(10 x) 1 m 36 s	12 m 56 s	(4 x) 1m 42 s	13 m 51 s
**Number of subjects**				
N iPD	24	46	46	72
N PD-LRRK2	1	5	3	2
N PD-GBA	3	8	7	7

For peer review only

N RBD	0	15	15	20
N RBD-GBA	0	1	1	1
N aGBA	0	8	8	8
N HC	19	26	26	25
N total	47	109	106	135

Legend: iPD = idiopathic Parkinson's patients; PD-LRRK2 = Parkinson's patients with mutation of the LRRK2 gene; PD-GBA = Parkinson's patients with mutation of the GBA gene; RBD = patients with REM sleep behavior disorder (RBD); RBD-GBA = RBD patients with pathogenic mutation of the GBA gene; aLRRK2 = asymptomatic carriers of a pathogenic mutation of the LRRK2 gene; aGBA = asymptomatic carriers of a pathogenic mutation of the GBA gene; HC = Healthy controls. [§] Two IR-SPGR images to assess B1 field inhomogeneity (72 slices, 2.3 x1.1 x 2.2 mm^3, TE 3.7 ms, TR 8 ms, TI 350 ms and 450 ms).

Supplementary figures

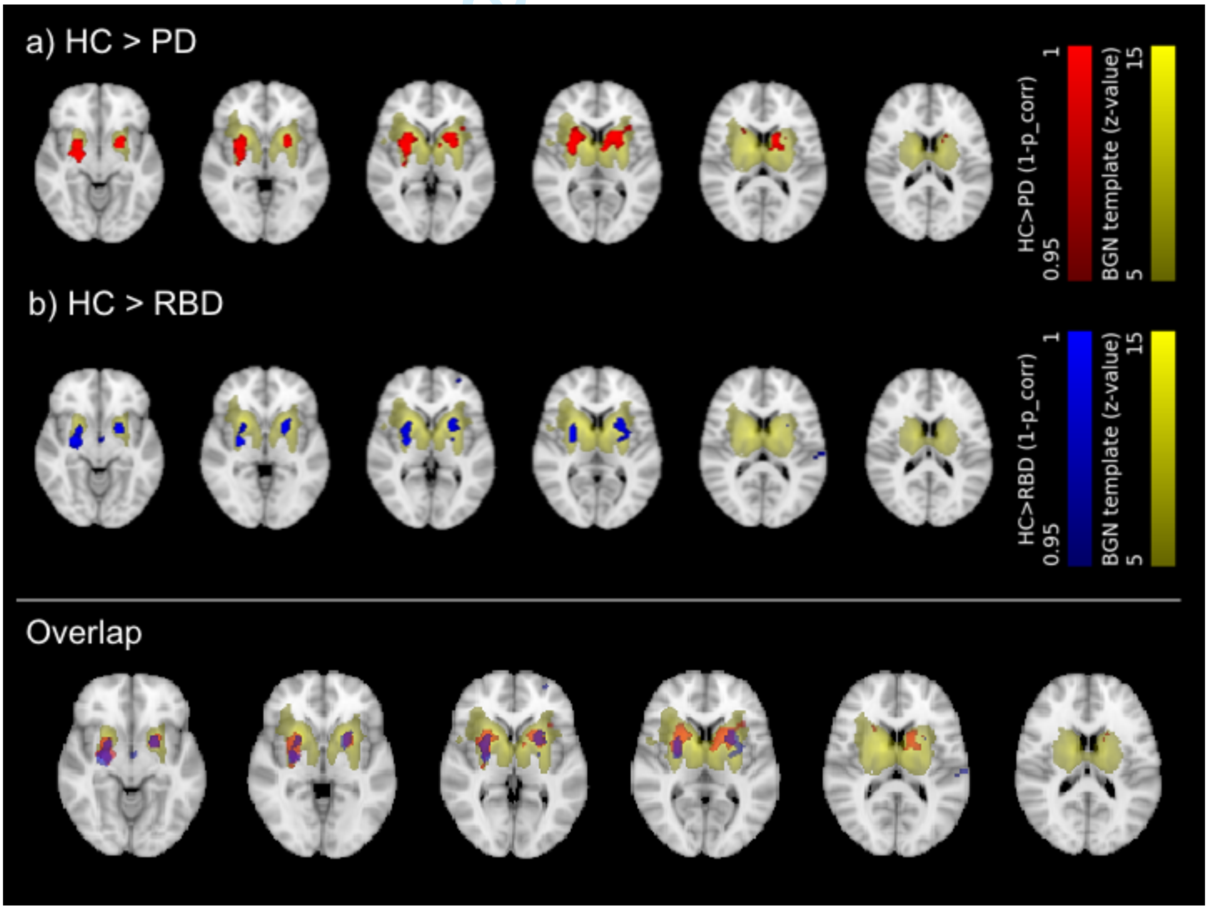

Figure S1. Resting state fMRI. Yellow underlay: basal ganglia network (BGN) template map, part of the template created with group ICA from 45 healthy controls external to the OPDC MRI sub-study (threshold of z=5 is applied for display purposes only; more details in [5], and

full template available online[6]). This was used in dual regression to extract subject-specific maps, which were then compared across groups. Results of the one-way ANOVA across 3 groups (n. PD = 103, n. RBD = 67, n. HC = 68) with post-hoc t-tests showed reduced functional connectivity in Parkinson's compared to controls (panel a, red) and reduced functional connectivity in RBD compared to controls (panel b, blue). Values displayed are 1-p>0.95, corresponding to p<0.05, corrected for multiple comparisons using threshold-free cluster enhancement. The bottom panel shows an overlap of the results. Full maps are available on NeuroVault (https://identifiers.org/neurovault.collection:5686). This is a replication of the analysis performed in [7] on a smaller sample (PD = 48, n. RBD = 26, n. HC = 23). These results are in line with the original study, although the effect size is smaller.

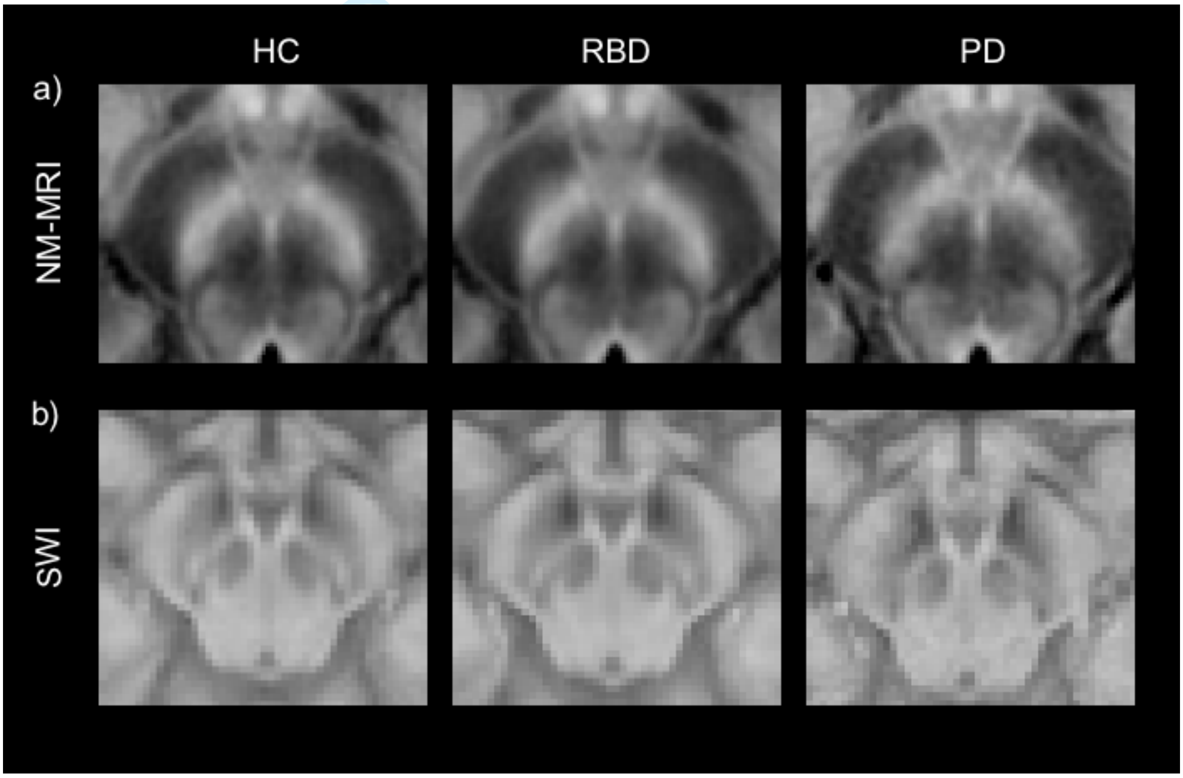

Figure S2. Novel sequences. In our cross-sectional sample, we observed a) progressive reduction of neuromelanin content in the substantia nigra pars compacta detected with NM-MRI (average maps from 25 HC, 42 RBD and 10 PD) and b) progressive reduction of the nigrosome-1 signal intensity (dorsal nigral hyperintensity - DNH - or 'swallow-tail sign') in SWI images (average maps from 32 HC, 44 RBD and 102 PD) from HC to RBD to manifest Parkinson's.

References

1. Vaillancourt DE, Spraker MB, Prodoehl J, et al. High-resolution diffusion tensor imaging in the substantia nigra of de novo Parkinson disease. *Neurology* 2009;72(16):1378-84. doi: 10.1212/01.wnl.0000340982.01727.6e
2. Deoni SC, Peters TM, Rutt BK. High-resolution T1 and T2 mapping of the brain in a clinically acceptable time with DESPOT1 and DESPOT2. *Magnetic resonance in medicine : official journal of the Society of Magnetic Resonance in Medicine / Society of Magnetic Resonance in Medicine* 2005;53(1):237-41. doi: 10.1002/mrm.20314
3. Baudrexel S, Nurnberger L, Rub U, et al. Quantitative mapping of T1 and T2* discloses nigral and brainstem pathology in early Parkinson's disease. *NeuroImage* 2010;51(2):512-20. doi: 10.1016/j.neuroimage.2010.03.005
4. Klein JC, Rolinski M, Griffanti L, et al. Cortical structural involvement and cognitive dysfunction in early Parkinson's disease. *NMR in biomedicine* 2018;31(4):e3900. doi: 10.1002/nbm.3900
5. Griffanti L, Rolinski M, Szewczyk-Krolikowski K, et al. Challenges in the reproducibility of clinical studies with resting state fMRI: An example in early Parkinson's disease. *NeuroImage* 2016;124(Pt A):704-13. doi: 10.1016/j.neuroimage.2015.09.021
6. OPDC MRI analysis [Available from: https://ora.ox.ac.uk/objects/uuid:8200af66-f438-4a7b-ad14-e8b032f0a9e7.
7. Rolinski M, Griffanti L, Piccini P, et al. Basal ganglia dysfunction in idiopathic REM sleep behaviour disorder parallels that in early Parkinson's disease. *Brain : a journal of neurology* 2016;139(Pt 8):2224-34. doi: 10.1093/brain/aww124

For peer review only

STROBE 2007 (v4) Statement—Checklist of items that should be included in reports of *cohort studies*

Section/Topic	Item #	Recommendation	Reported on page #
**Title and abstract**	1	(*a*) Indicate the study's design with a commonly used term in the title or the abstract	2
		(*b*) Provide in the abstract an informative and balanced summary of what was done and what was found	2
**Introduction**			
Background/rationale	2	Explain the scientific background and rationale for the investigation being reported	4
Objectives	3	State specific objectives, including any prespecified hypotheses	5
**Methods**			
Study design	4	Present key elements of study design early in the paper	5-7
Setting	5	Describe the setting, locations, and relevant dates, including periods of recruitment, exposure, follow-up, and data collection	7
Participants	6	(*a*) Give the eligibility criteria, and the sources and methods of selection of participants. Describe methods of follow-up	5-7
		(*b*) For matched studies, give matching criteria and number of exposed and unexposed	N/A
Variables	7	Clearly define all outcomes, exposures, predictors, potential confounders, and effect modifiers. Give diagnostic criteria, if applicable	5-7
Data sources/ measurement	8*	For each variable of interest, give sources of data and details of methods of assessment (measurement). Describe comparability of assessment methods if there is more than one group	7-13
Bias	9	Describe any efforts to address potential sources of bias	7-13
Study size	10	Explain how the study size was arrived at	5
Quantitative variables	11	Explain how quantitative variables were handled in the analyses. If applicable, describe which groupings were chosen and why	7-13
Statistical methods	12	(*a*) Describe all statistical methods, including those used to control for confounding	N/A
		(*b*) Describe any methods used to examine subgroups and interactions	N/A
		(*c*) Explain how missing data were addressed	N/A
		(*d*) If applicable, explain how loss to follow-up was addressed	N/A
		(*e*) Describe any sensitivity analyses	N/A
**Results**			

For peer review only

Participants	13*	(a) Report numbers of individuals at each stage of study—eg numbers potentially eligible, examined for eligibility, confirmed eligible, included in the study, completing follow-up, and analysed	Tables
		(b) Give reasons for non-participation at each stage	5,6
		(c) Consider use of a flow diagram	N/A
Descriptive data	14*	(a) Give characteristics of study participants (eg demographic, clinical, social) and information on exposures and potential confounders	Table 1
		(b) Indicate number of participants with missing data for each variable of interest	Table 2,3
		(c) Summarise follow-up time (eg, average and total amount)	N/A
Outcome data	15*	Report numbers of outcome events or summary measures over time	N/A
Main results	16	(*a*) Give unadjusted estimates and, if applicable, confounder-adjusted estimates and their precision (eg, 95% confidence interval). Make clear which confounders were adjusted for and why they were included	N/A
		(*b*) Report category boundaries when continuous variables were categorized	N/A
		(*c*) If relevant, consider translating estimates of relative risk into absolute risk for a meaningful time period	N/A
Other analyses	17	Report other analyses done—eg analyses of subgroups and interactions, and sensitivity analyses	N/A
**Discussion**			
Key results	18	Summarise key results with reference to study objectives	13
**Limitations**			
Interpretation	20	Give a cautious overall interpretation of results considering objectives, limitations, multiplicity of analyses, results from similar studies, and other relevant evidence	13-17
Generalisability	21	Discuss the generalisability (external validity) of the study results	13-17
**Other information**			
Funding	22	Give the source of funding and the role of the funders for the present study and, if applicable, for the original study on which the present article is based	22

*Give information separately for cases and controls in case-control studies and, if applicable, for exposed and unexposed groups in cohort and cross-sectional studies.

Note: An Explanation and Elaboration article discusses each checklist item and gives methodological background and published examples of transparent reporting. The STROBE checklist is best used in conjunction with this article (freely available on the Web sites of PLoS Medicine at http://www.plosmedicine.org/, Annals of Internal Medicine at http://www.annals.org/, and Epidemiology at http://www.epidem.com/). Information on the STROBE Initiative is available at www.strobe-statement.org.

For peer review only
